# A thraustochytrid-specific lipase/phospholipase with unique positional specificity contributes to microbial competition and fatty acid acquisition from the environment

**DOI:** 10.1038/s41598-019-52854-7

**Published:** 2019-11-08

**Authors:** Yohei Ishibashi, Keisuke Aoki, Nozomu Okino, Masahiro Hayashi, Makoto Ito

**Affiliations:** 1 Department of Bioscience and Biotechnology, Graduate School of Bioresource and Bioenvironmental Sciences, Kyushu University, 744 Moto-oka, Nishi-ku Fukuoka, 819-0395 Japan; 2 Department of Marine Biology and Environmental Sciences, Faculty of Agriculture, University of Miyazaki, 1-1 Gakuen-kibanadai-nishi, Miyazaki, 889-2192 Japan; 3 Innovative Bio-architecture Center, Kyushu University, 744 Moto-oka, Nishi-ku Fukuoka, 819-0395 Japan

**Keywords:** Lipids, Environmental microbiology, Biochemistry, Ecology, Microbiology

## Abstract

Thraustochytrids are heterotrophic marine protists that are considered as important decomposers in the marine ecosystem; however, how they digest and uptake lipid nutrients from the environment is largely unknown. Genomic clustering analysis using thraustochytrid draft genome databases revealed that novel proteins with a Lipase_3 domain are commonly present in thraustochytrids, including *Aurantiochytrium limacinum*. After heterologous expression and His tag-based purification, protein ID: 145138 was identified as lipase/phospholipase capable of hydrolyzing triacylglycerol (TG) and phosphatidylcholine (PC). 145138 was secreted into the medium, and deletion of the 145138 gene in *A. limacinum* reduced the degradation of extracellular lipids. Fatty acids generated by 145138 were reused for the biosynthesis of PC and TG, and 145138 allowed *A. limacinum* to survive in the medium containing TG as a sole carbon source. 145138 hydrolyzed all the acyl-ester linkages of TG; however, the enzyme showed strict positional specificity toward phospholipids, generating 2-acyl lysophospholipids. The 2-acyl lysophospholipids showed stronger antimicrobial activity compared with 1-acyl lysophospholipids. These results suggested that 145138 is a bifunctional enzyme that contributes to the acquisition of lipid nutrients from the environment, as well as to generate antimicrobial lysophospholipids that are beneficial for competition with bacteria over lipid nutrients in the marine environment.

## Introduction

Thraustochytrids are eukaryotic marine protists, including the typical genera *Aurantiochytrium*, *Aplanochytrium*, *Schizochytrium*, *Thraustochytrium*, and *Parietichytrium*, which belong to the Stramenopiles, class Labyrinthulomycetes, family Thraustochytriaceae^1,2^. These organisms are commonly found in marine and brackish environments and are considered as an important decomposer in marine ecosystem^3,4^, probably affecting the microbial food web and carbon cycle in the ocean. They are considered to have lost chloroplasts during evolution; therefore, they are obligate heterotrophs. Thraustochytrids obtain nutrition from the environment^5–7^. However, there is limited knowledge about how thraustochytrids uptake lipid nutrition from the environment and what molecular machinery is involved in this process. Identification of secretory lipid-decomposing enzymes may help to understand these issues and the contribution of thraustochytrids to the lipid-derived organic carbon cycle in the marine environment.

Thraustochytrids have recently received increasing attention from academic as well as industrial researchers, because they produce huge amounts of n-3 polyunsaturated fatty acids (n-3PUFA), such as docosahexaenoic acid (DHA; 22:6n-3), in conventional medium containing glucose as a carbon source^8–10^. Thraustochytrids are known as oleaginous microorganisms that accumulate n-3PUFAs and palmitic acid mainly as acyl chains of triacylglycerol (TG) in lipid droplets (LDs) and phosphatidylcholine (PC) in cellular membranes.

In general, oleaginous microorganisms such as *Yarrowia lipolytica* have many secretory lipases that might be utilized for nutrient acquisition from surrounding lipids and are attributed to their obese phenotype^11^. Thus, we considered that thraustochytrids should have secretory lipases, and we tried to identify secretory lipase genes from a draft genome database of thraustochytrids; however, no homologous genes were found in BLAST searches using known secretory lipases as query sequences. On the other hand, it was reported that thraustochytrids secrete enzymes including lipase^12,13^. These results suggested that thraustochytrids may have novel extracellular lipases, for which sequences have not yet been reported.

*Phaeodactylum tricornutum* and *Thalassiosira pseudonana* are autotrophic marine diatoms belonging to the Stramenopiles, the same as the thraustochytrids. The Joint Genome Institute (JGI) provides genomic databases for Stramenopiles, including *Aurantiochytrium limacinum, P. tricornutum*, and *T. pseudonana* in Genome Portal^14,15^. We predicted that genes for secretory lipases should exist only in heterotrophic thraustochytrids, and comparative genomic analysis between thraustochytrids and diatoms might reveal the thraustochytrid-specific genes. As expected, thraustochytrid-specific genes were successfully extracted from the databases by comparative gene clustering, and several unique lipase-like genes were found in the genome of *A. limacinum* in this study. Here, we report the isolation and characterization of a novel lipase (named 145138 from its protein ID assignment in JGI), which shows a novel positional specificity toward TG and phospholipids. This research provides insights into the biological significance of 145138 in thraustochytrids for lipid uptake from the marine environment.

## Results

### Lipase-like genes found in genome databases of thraustochytrids

**Figure 1:**
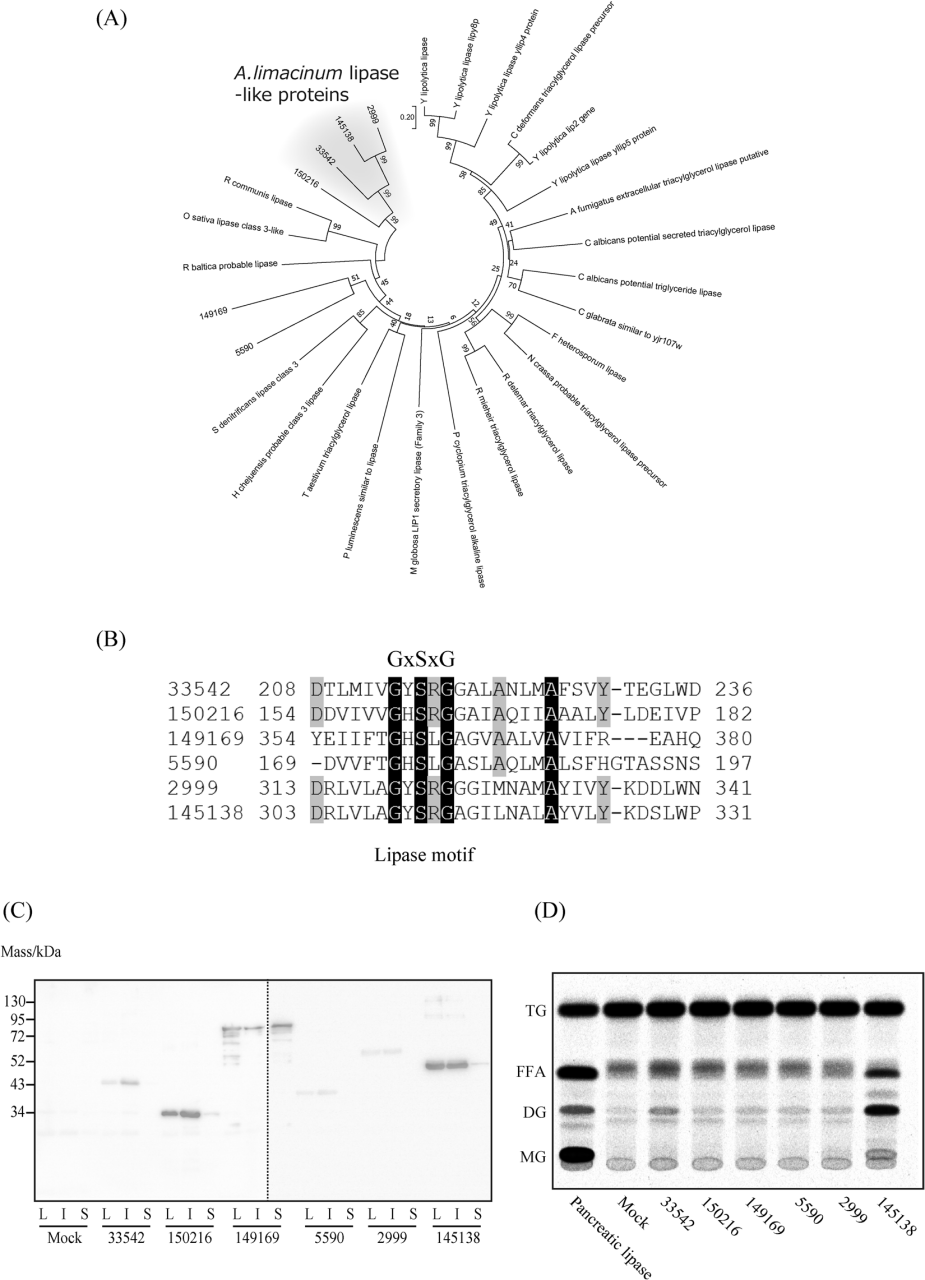
Phylogenic analysis and heterologous expression of lipase-like genes of thraustochytrid. (**A**) Phylogenic tree of class 3 lipases and lipase-like proteins of A. *limacinum*. Amino acid sequences of several class 3 lipases and lipase-like genes found in thraustochytrid were reconstructed using the neighbor-joining method. The evolutionary distances were computed using the Poisson correction method and shown as the number of amino acid substitutions per site. (**B**) Alignment of the primary sequences around the lipase motif (GXSXG) of lipase-like gene products (Protein ID: 33542, 150216, 149169, 5590, 2999, and 145138) of *A. limacinum*. Amino acid sequences of lipase-like proteins were aligned using MUSCLE. Identical residues are shown as white letters on a black background. (**C**) Western blotting analysis showing the expression of lipase-like gene products of *A. limacinum* using an *E. coli* expression system. Recombinant proteins were detected from cell lysates (L), insoluble fractions (I), and soluble fractions (S) by western blotting using an anti-6× His antibody. Two different PVDF membranes were combined to show all of the lipase-like gene products. (**D**) Lipase activity of lipase-like proteins expressed in *E. coli*. Cell lysates were incubated with ^14^C-labeled triolein for 30 min at 25 °C, then the reaction mixtures were applied to TLC plates that was developed with hexane/diethyl ether/acetic acid = 50/50/1 (v/v/v) as a developing solvent. TLC autoradiography of TG, DG, MG, and FFA containing ^14^C-oleic acid was quantified by Typhoon FLA 9500 (GE Healthcare). Pancreatic lipase (25 μg) was used as a control.

JGI provides information about the clustering of genes in organisms belonging to the Stramenopiles, which facilitates the identification of mutual relationships among genes. We searched for genes that were specifically distributed in thraustochytrids using comparative clustering analysis. As a result, we found several thraustochytrid-specific, novel, lipase-like gene products, protein IDs: 2999, 145138, 33542, 150216, 149169, and 5590, all of which contained a Lipase_3 (PF01764) domain, in the draft genome database of *A. limacinum* (Fig. 1A). Multiple sequence alignments showed that these lipase-like gene products had no similarity with previously known lipases except for the Lipase_3 (PF01764) domain, thereby forming the unique branch of class 3 lipases in the phylogenic tree (Fig. 1A).

### Lipase activity of lipase-like gene products of A. limacinum expressed in *E. coli*

In general, lipases possess a pentapeptide GXSXG motif, in which serine acts as the active site^16^. This lipase motif was conserved in 6 lipase-like gene products in *A. limacinum* (Fig. 1B). However, the overall putative amino acid sequences of these gene products showed very low identity with lipases reported so far. Six lipase-like genes were cloned using cDNAs from *A. limacinum* mh0186, inserted into the pCold vectors, and expressed in *E. coli* as N-terminal 6× His tagged proteins. Western blotting analysis revealed that all of the lipase-like proteins were successfully expressed in *E. coli*; however, protein IDs 5590 and 2999 were only detected in the insoluble fractions (Fig. 1C). Lipase activity was measured using radioisotope-labeled triolein (TG 54:3, 18:1/18:1/18:1) as a substrate and lysate as an enzyme source. Free fatty acids (FFAs), diacylglycerol (DG), and monoacylglycerol (MG) were generated from TG by the action of 145138 and pancreatic lipase (control) (Fig. 1D). TG was also hydrolyzed by 33542 to produce FFA and DG; however, MG was not detected by autoradiography in the conditions used (Fig. 1D). 150216 and 149169 did not show lipase activity, although the corresponding gene products were detected in the soluble fraction (Fig. 1D). Thus, we conducted more-detailed investigations using 145138 in this study. The amino acid sequence of 145138 showed very low identity (13.6%) with that of *Rhizomucor miehei* lipase, which is a representative lipase containing the Lipase_3 domain^17^. No sequences homologous to 145138 were found in the Stramenopiles genome databases other than in thraustochytrids, suggesting that 145138 is a thraustochytrid-specific lipase.

### Effect of the disruption of 145138 in A. limacinum on extracellular lipids

**Figure 2:**
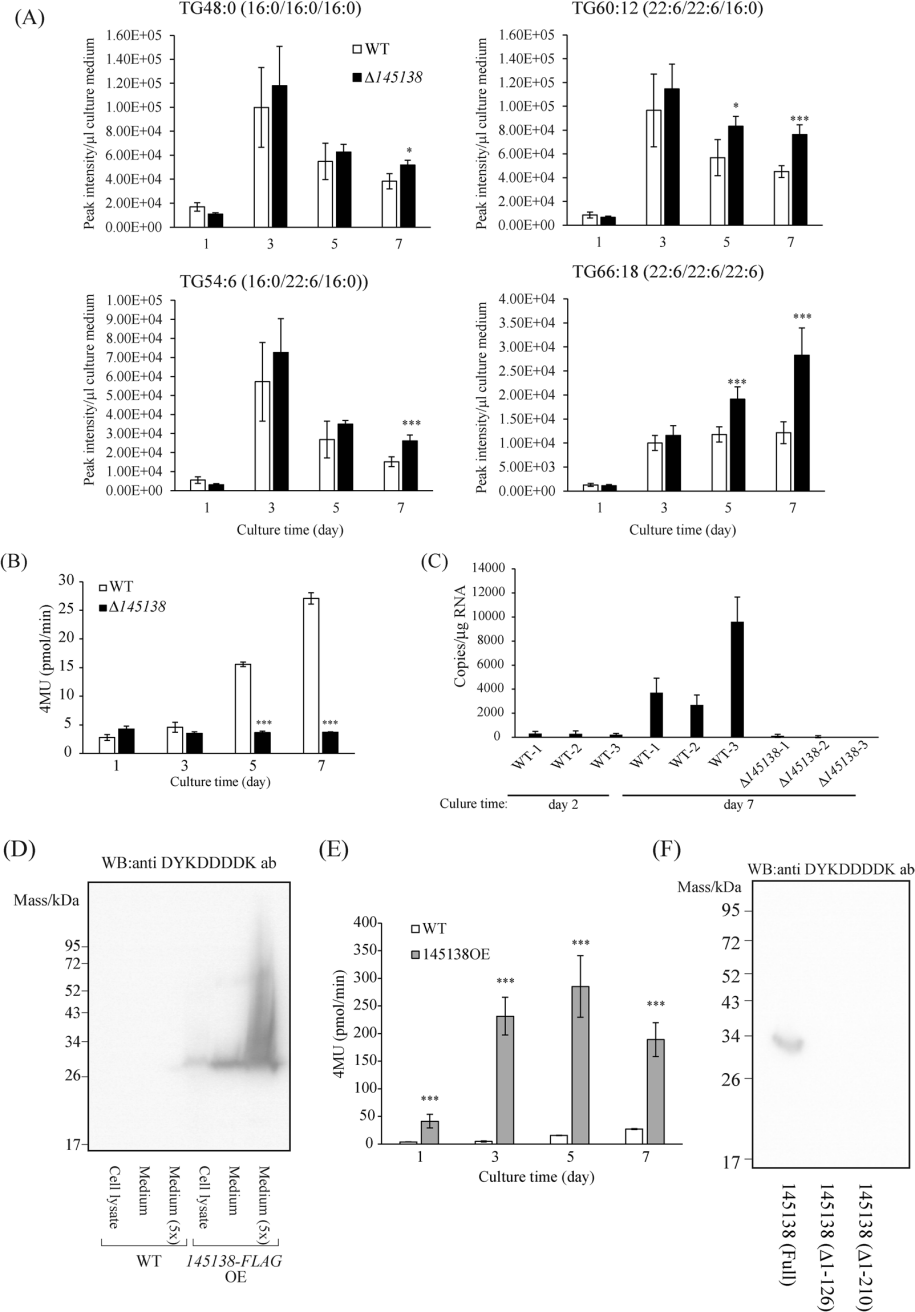
Involvement of 145138 in the extracellular lipid degradation. (**A**) Amounts of extracellular TGs. Culture supernatants of WT and Δ145138 were collected by centrifugation, and lipids were extracted to subject to MS analysis. Major molecular species, TG48:0, TG54:6, TG60:12, and TG66:18, in WT (white bars) and Δ145138 (black bars) were measured by MRM analysis using LC-ESI MS/MS. (**B**) Lipase activities in the medium of WT and Δ145138. The culture supernatant was collected by centrifugation from 1-, 3-, 5-, and 7-day cultures of WT and Δ145138. Lipase activities were measured by using 4MU-palmitic acid as a substrate. (**C**) mRNA expression levels of 145138. Cells were harvested from 2- and 7-day cultures of WT and Δ145138. A standard curve to determine the copy numbers of 145138 was made from a plasmid containing 145138. (**D**) Western blotting analysis showing the localization of 145138-FLAG. After cultivation for 3 days, the culture supernatant and cells of WT and 145138-FLAG-OE were separated by centrifugation. Cell lysate, culture supernatant, and 5-times concentrated supernatant were subjected to western blotting using an antibody against DYKDDDDK. (E) Lipase activities in the supernatant of WT and 145138-FLAG-OE. Culture supernatants were collected from 1-, 3-, 5-, and 7-day cultures of WT and 145138-FLAG-OE. Lipase activities were measured by using 4MU-palmitic acid as a substrate. (**F**) Western blotting analysis showing the expression level of full length 145138 [145138 (Full)] and N-terminal deleted mutants, 145138 (Δ1–126) and 145128 (Δ1–210) in the culture medium. All constructs were expressed as C-terminal FLAG-fused proteins. Error bars represent means ± S.D. of three separate experiments.

To investigate the function of 145138, we constructed a 145138-disrupted mutant (Δ145138) by homologous recombination using a hygromycin B resistance gene (HygR) as a selectable marker (Fig. S1A,B)^18,19^. Cell growth of Δ145138 was almost the same as that of wild-type (WT) during the culture period when cell growth was examined by measuring optical density (OD) at 600 nm and glucose consumption (Fig. S1C,D), indicating that 145138 is not an essential gene for *A. limacinum* in normal culture medium in which the carbon source is glucose. The effects of disruption of 145138 on the amounts of major TG species (TG48:0, TG54:6, TG60:12, and TG66:18) in *A. limacinum*^9^ were examined because 145138 seemed to be a TG lipase (Fig. 1D). The content of cellular TG increased until stationary phase and then decreased gradually (Fig. S1E), suggesting that cellular TG was synthesized in logarithmic growth phase and degraded by lipase(s) after the consumption of glucose in the medium. However, 145138 was not likely to be responsible for the intracellular TG metabolism because cellular TG levels were almost the same between WT and Δ145138 during the cultivation period (Fig. S1E). On the other hand, extracellular TG increased significantly in Δ145138 at days 5 and 7, compared with WT (Fig. 2A). This result indicted that a considerable amount of TG was released into the culture medium after the stationary phase, and 145148 could degrade the extracellular TG, suggesting that 145138 is secretory lipase that may be involved in the degradation of environmental lipids.

### Localization of 145138

Lipase activities in the medium of WT and Δ145138 cultures were measured using 4-methylumbelliferyl (4MU)-palmitate as a substrate^20^. Extracellular lipase activity in the WT increased significantly at days 5 and 7, indicating that the lipase was released into the culture medium of *A. limacinum* after the consumption of glucose in the medium (Fig. 2B, Fig. S1D). We found that the extracellular lipase activity of Δ145138 significantly lower than WT at days 5 and 7 (Fig. 2B). The expression of 145138 mRNA was low in WT at day 2 and increased at day 7 (Fig. 2C). It was confirmed that 145138 mRNA was not expressed in Δ145138. These results indicated that *145138* is expressed after the stationary phase and then secreted into the medium. To determine the localization of 145138, C-terminal FLAG-tagged 145138 was expressed in *A. limacinum* using a thraustochytrid-oriented transformation system^18^ (Fig. S2A). The 145138 overexpression (145138OE) cell line of *A. limacinum* did not exhibit altered cell growth in glucose-containing medium (Fig. S2B). Western blotting using an anti-FLAG antibody revealed that most 145138-FLAG was secreted into the medium when expressed in *A. limacinum*, although the FLAG-tag protein was detected in both the cell lysate and culture medium (Fig. 2D). In agreement with this result, lipase activity increased significantly in the culture medium of 145138OE compared with the WT (Fig. 2E). These results indicated that 145138 is an extracellular lipase, which is possibly involved in the extracellular metabolism of lipids. The molecular mass of 145138 expressed in *A. limacinum* was calculated to be 30 kDa by SDS-PAGE (Fig. 2D), which was much smaller than that expressed in *E. coli* (Fig. 1C) or the molecular mass (46.5 kDa) estimated from the deduced amino acid sequence of 145138. This result suggested that an alternative translational start codon was used for the expression of 145138, or the N-terminal region could be processed before or after secretion, because the FLAG-tag located at the C-terminus of 145138 was retained after secretion. To validate these possibilities, two variants 145138(Δ1–126) and 145138(Δ1–210) with different initiation codons were expressed in the *A. limacinum* Δ145138 mutant (Fig. S2C). Although 145138 (Full) was detected in the medium, the expression of 145138 (Δ1–126) and 145138 (Δ1–210) was not observed by western blotting using an anti-FLAG antibody (Fig. 2F). We found that not only extracellular expression but also intracellular expression was not detected in 145138 (Δ1–126) and 145138 (Δ1–210) (Fig. S2D). There results indicated that protein translation of 145138 requires the initiation codon of 145138 (Full) (Fig. S2C), and protein truncation appeared to occur after expression as 145138 (Full) in the secretion process. Secretory proteins generally contain a signal sequence at the N-terminus^21^. However, no known signal sequence was found in the N-terminal region of 145138, and it was predicted to be a cellular protein that is likely to be a type II transmembrane protein^22,23^ (Fig. S2E).

### Lipid compositions of the medium of 145138OE

**Figure 3:**
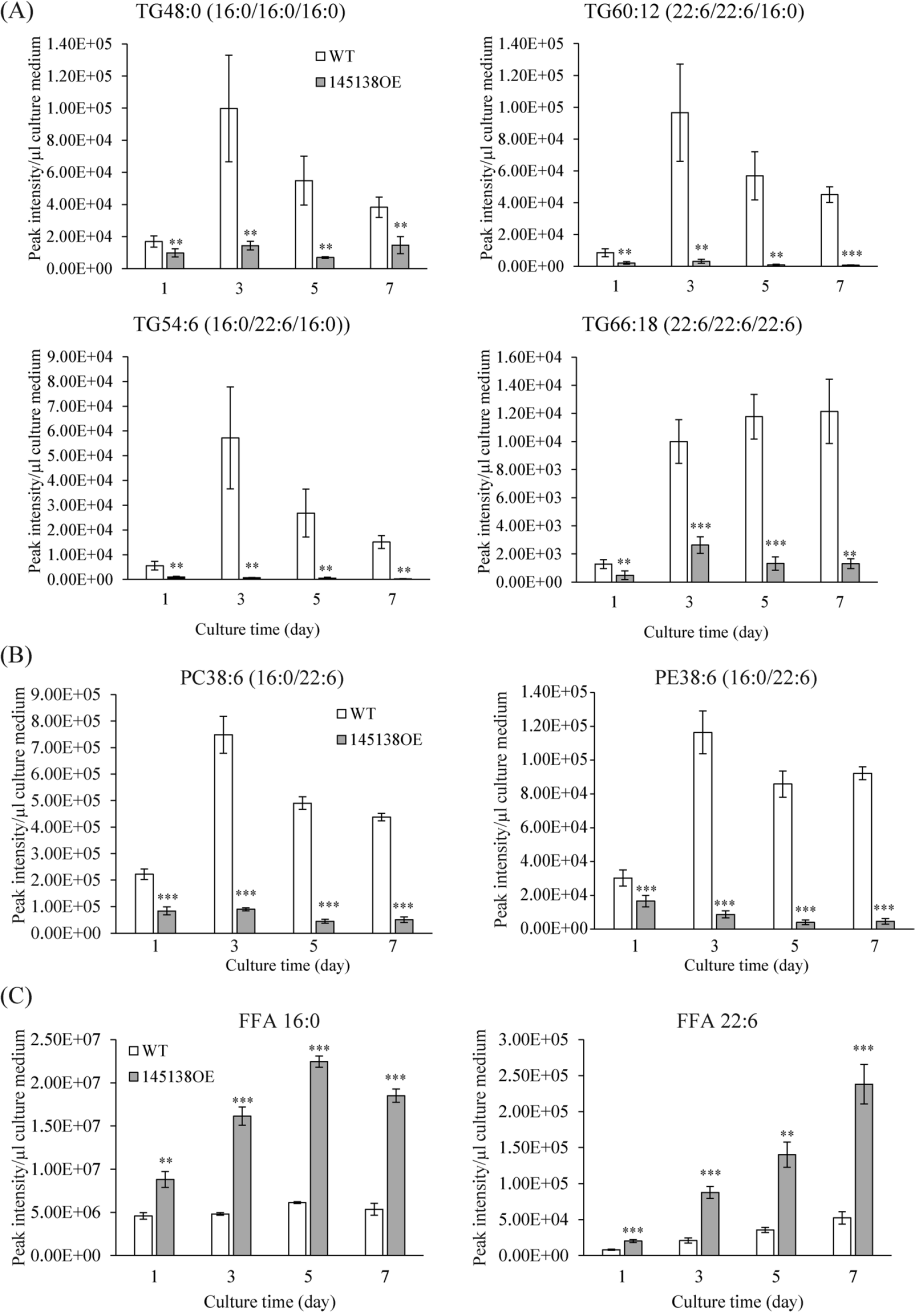
Extracellular lipid compositions of WT and 145138OE. Quantification of extracellular lipids of WT and 145138OE using LC-ESI MS/MS. (**A**) TG48:0, TG54:6, TG60:12, and TG66:18. (**B**) PC38:6 and PE38:6. (**C**) Free palmitic acid (16:0) and DHA (22:6). Supernatants of WT and 145138-FLAG-OE were harvested by centrifugation from 1-, 3-, 5-, and 7-day culture samples. Error bars represent means ± S.D. of four separate experiments.

Consistent with an increase in lipase activity in the 145138OE line, TG levels decreased markedly in the culture medium after overexpression during cultivation (Fig. 3A). A decrease in TG was observed for all molecular species of TG examined (Fig. 3A). Of note, the amount of PC and phosphatidylethanolamine (PE) also decreased in the medium in the 145138OE line (Fig. 3B). Along with a decrease in TG and phospholipids levels, the FFA level increased in the medium of the 145138OE line (Fig. 3C). These results indicated that secretory lipase 145138 hydrolyzes not only neutral lipids but also phospholipids in the culture medium.

### General properties of 145138 expressed in A. limacinum

To examine the enzymatic properties of 145138, we purified recombinant 145138 from the medium of 6× His-tagged 145138OE using an Ni-Sepharose 6 Fast Flow column and a HiTrap desalting column. The final preparation showed a 30 kDa single protein band on SDS-PAGE after staining with Coomassie Brilliant Blue (CBB) (Fig. S3A left) and the anti-6× His tag antibody (Fig. S3A right). In a typical experiment, 1 mg of purified recombinant 145138 was obtained from a 1 L culture of 6× His-tagged 145138OE. Protein sequence analysis revealed that the sequence at the N-terminal of the purified 145138 is ALSSS (Fig. S2C), indicating that the cleavage occurs between L159 and A160 during the secretion process (Fig. S2C). The general properties of 145138 were characterized using the purified enzyme. Maximal activity was observed at around pH 8 and 35 °C (Fig. S3B C). Lipase activity was strongly enhanced by adding methanol and ethanol at concentrations of 25% (Fig. S3D). 145138 was activated by Ca^2+^ and Mg^2+^ ions (Fig. S3E), and the maximum activity was observed by adding Ca^2+^ to more than 12.5 mM (Fig. S3F). On the other hand, the activity of 145138 decreased drastically after the addition of EDTA (Fig. S3E,F), indicating that Ca^2+^ was indispensable for the activity of 145138. Co^2+^, Cu^2+^, Hg^2+^, Mn^2+^, Ni^2+^, Fe^3+^, and Zn^2+^ at concentrations of 5 mM strongly inhibited the activity of 145138 (Fig. S3E). We found that the activity of 145138 was stable even with a concentration of 2.5 M NaCl (Fig. S3G), indicating that 145138 is a halotolerant lipase.

### Substrate specificity of 145138

**Figure 4:**
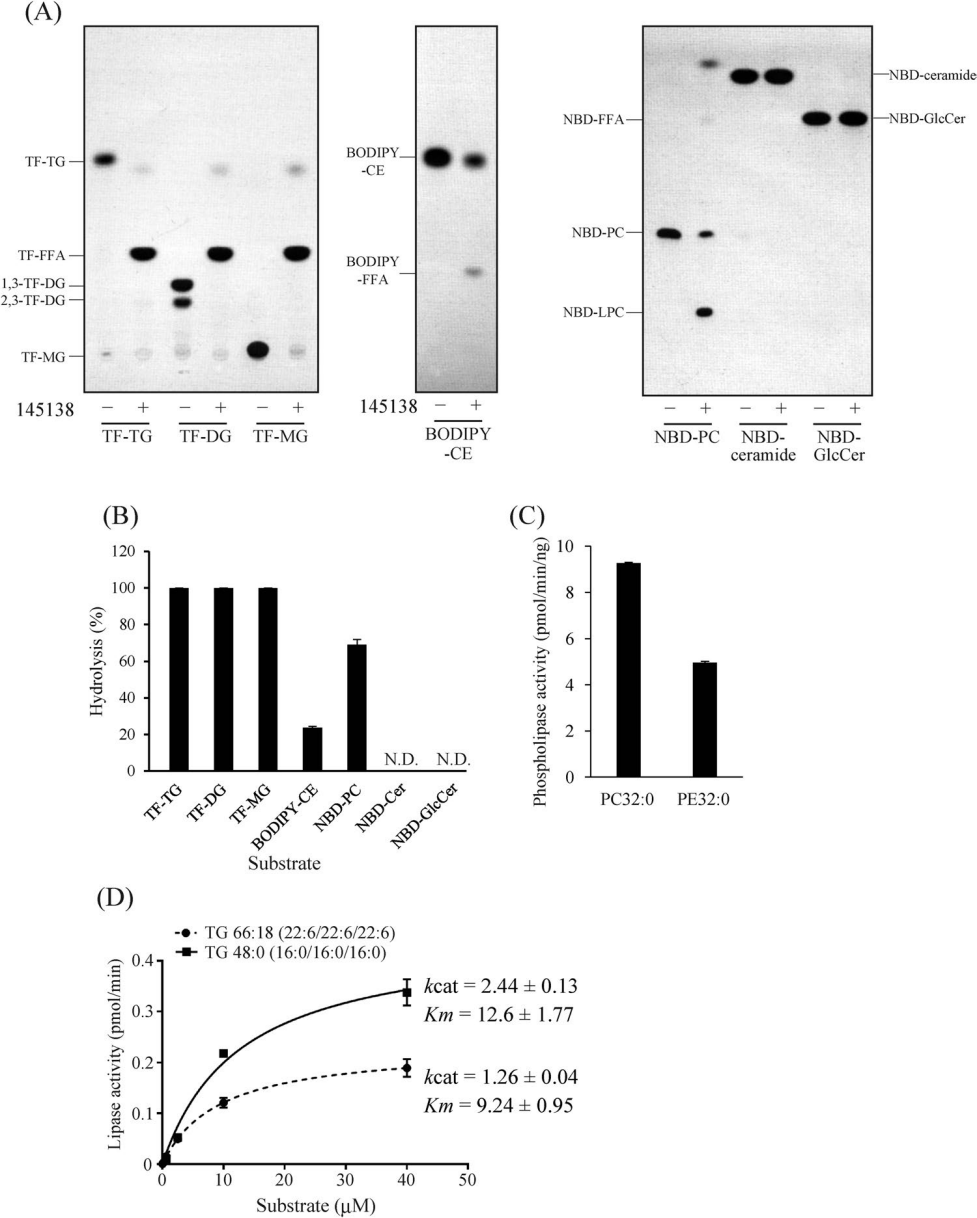
Substrate specificities of recombinant 145138. (**A**) TLC analysis showing the hydrolysis of fluorescent lipid substrates using recombinant 145138. One hundred pmol of each substrate was incubated without (−) or with (+) 145138 at 37 °C for 60 min. TLC plates were developed with hexane/diethyl ether/acetic acid (50/50/1, v/v/v) for TF-TG, TF-DG, TF-MG, and BODIPY-CE and chloroform/methanol/water (65/35/8, v/v/v) for NBD-PC, NBD-ceramide, and NBD-GlcCer. (**B**) Quantification of hydrolysis extents of fluorescent lipid substrates using recombinant 145138. Fluorescence intensities of each substrate and product were quantified using a fluorescence chromatoscanner. The extent of hydrolysis of fluorescent lipids was calculated as follows: hydrolysis (%) = (peak area for product) × 100/(peak area for product + peak area for remaining substrate). (**C**) Hydrolytic extent of PC32:0 and PE32:0 of 145138. One nmol each of PC32:0 and PE32:0 was incubated with 145138, and the remaining substrates were quantified using LC-ESI MS/MS. (**D**) Michaelis–Menten plots of the hydrolysis of TG66:18 and TG48:0 by recombinant 145138. Several concentrations of TG66:18 and TG48:0 were incubated with 145138, and the remaining substrates were quantified using LC-ESI MS/MS. Kinetic parameters (*k*cat and *Km*) for each substrates were shown in the graph. Error bars represent means ± S.D. of three separate experiments. N.D., not detected.

To determine the substrate specificity of 145138, various fluorescent lipids were used as substrates. All lipids underwent labeling with a fluorescent tag at the methyl end of the fatty acid moiety but the type of fluorescent compound depended on the lipid (Fig. S4). It was confirmed using thin-layer chromatography (TLC) that commercial 1,3-TF-DG contained 1,3-TF-DG as well as 2,3-TF-DG (or 1,2-TF-DG) (Fig. 4A, Fig. S4). It is possible that 2,3-TF-DG is generated from 1,3-TF-DG by an acyl-migration reaction from the *sn*-1 or 3 to the *sn*-2 position in the glycerol backbone^24^. We found that fluorescent TG, DG, and MG were completely hydrolyzed by 145138 to generate fluorescent FFA (TF-FFA) after a 60-min incubation (Fig. 4A). In the same conditions, NBD-PC was hydrolyzed by 145138 to generate NBD-lysophosphatidylcholine (LPC) but not NBD-FFA (Fig. 4A), suggesting the enzyme specifically cleaved the ester linkage at *sn*-1 of PC because NBD is conjugated to the fatty acid at *sn*-2 (Fig. S4). BODIPY-cholesterol ester (CE) was hydrolyzed by 145138, but the reaction was very slow (Fig. 4A). Sphingolipids such as glucosylceramide and ceramide were completely resistant to hydrolysis by 145138 (Fig. 4A). The extent of hydrolysis of each fluorescent lipid by 145318 was quantified using a fluorescent TLC chromatoscanner, and PC32:0 and PE32:0 were quantified by mass spectrometric analysis. As a result, 145138 efficiently hydrolyzed neutral glycerolipids, such as TG, DG, and MG, and glycerophospholipids, such as PC and PE (Fig. 4B,C). On the other hand, CE was relatively resistant to hydrolysis, and sphingolipids were not hydrolyzed by 145138 (Fig. 4B). These results indicated that 145138 is a lipase/phospholipase that acts on the acyl-ester linkage of glycerolipids. Next, we examined the substrate specificity of 145138 toward the fatty acid moiety of TG using TG 48:0 (16:0/16:0/16:0) and TG 66:18 (22:6/22:6/22:6). TG 48:0 was hydrolyzed by 145138 approximately 2 times faster than TG66:18, indicating that 145138 prefers palmitic acid (16:0) rather than DHA (22:6) in the condition used (Fig. 4D).

### Positional specificity (regiospecificity) of 145138 to TG

**Figure 5:**
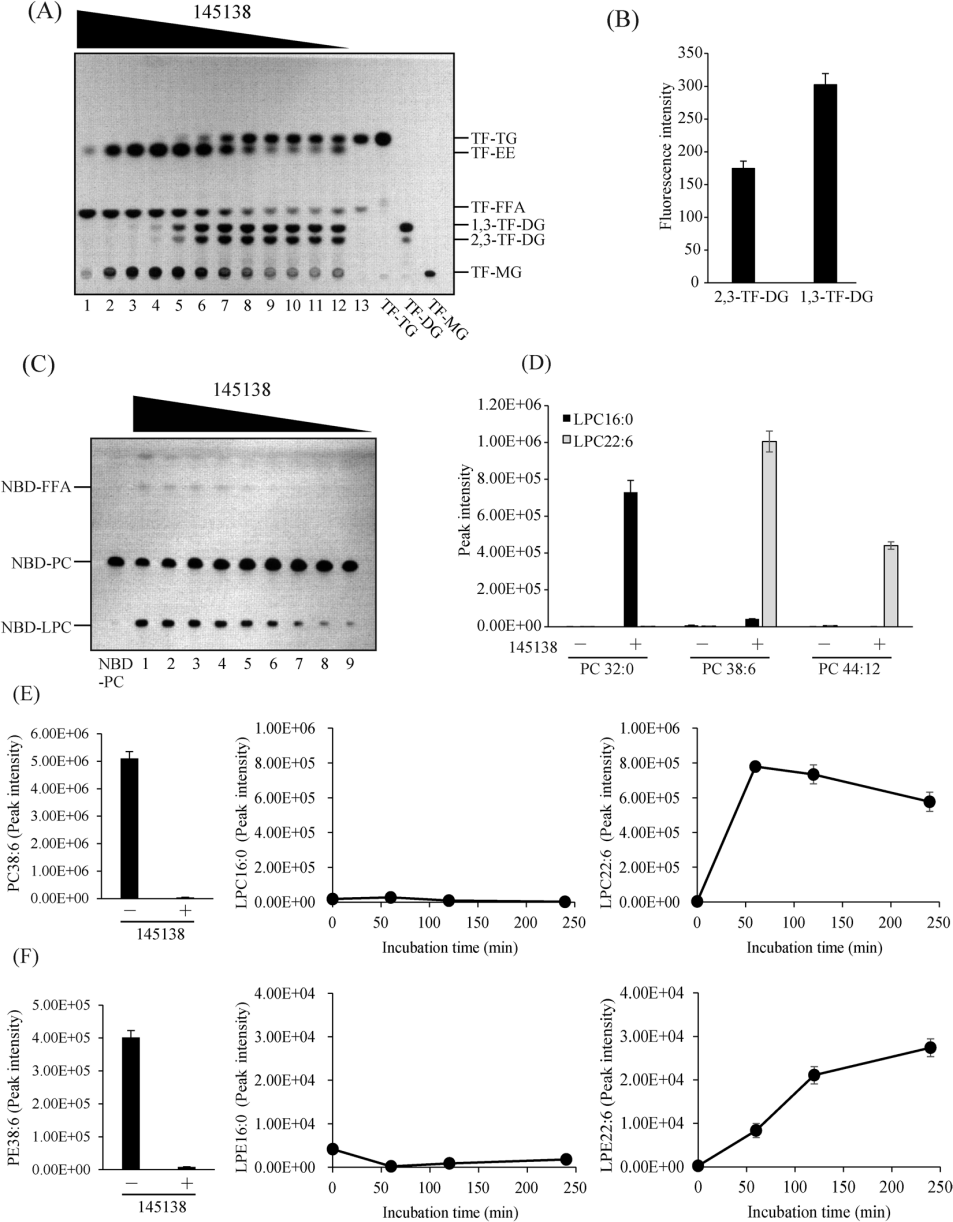
Positional specificities of 145138 on TG and phospholipids. (**A**) TLC analysis showing the release of products from TF-TG by incubation with different amounts of the recombinant 145138 (lane 1, 12.5 ng; lane 2, 6.3 ng; lane 3, 3.1 ng; lane 4, 1.6 ng; lane 5, 0.8 ng; lane 6, 0.4 ng; lane 7, 0.2 ng; lane 8, 0.1 ng; lane 9, 50 pg; lane 10, 25 pg; lane 11, 12.5 pg; lane 12, 6 pg, lane 13, without enzyme) at 37 °C for 20 min. (**B**) Quantification of 2,3-TF-DG and 1,3-TF-DG generated from TF-TG with 0.2 ng of recombinant 145138. The fluorescence intensities of 2,3-TF-DG and 1,3-TF-DG were measured using a chromatoscanner. (**C**) TLC showing the products generated from NBD-PC using different amounts of 145138 (lane 1, 12.5 ng; lane 2, 6.3 ng; lane 3, 3.1 ng; lane 4, 1.6 ng; lane 5, 0.8 ng; lane 6, 0.4 ng; lane 7, 0.2 ng; lane 8, 0.1 ng; lane 9, 50 pg) at 37 °C for 20 min. (**D**) Quantification of LPC16:0 and LPC22:6 generated from PC32:0, PC38:6, and PC44:12. LPCs were quantified using LC-ESI MS/MS after incubation without (−) or with (+) 145138. (**E**) Time courses for the peak intensities of LPC16:0 (middle panel) and LPC22:6 (right panel) generated from PC 38:6 (left panel) by the action of 145138. (**F**) Time courses for the peak intensities of LPE16:0 (middle panel) and LPE22:6 (right panel) generated from PE 38:6 (left panel) by the action of 145138. Error bars represent means ± S.D. of three separate experiments.

There are two types of TG lipase, one hydrolyzes acyl ester linkages at *sn*-1 and *sn*-3 but not *sn*-2 (*sn*-1/3-specific lipase), and the other shows no positional specificity (non-specific lipase), hydrolyzing the acyl ester linkages randomly^24,25^. The positional specificity of 145138 was examined using fluorescent TG (TF-TG) labeled with fluorescent tag at *sn*-3 (Fig. S4) and different concentrations of 145138 (Fig. 5A, concentration gradient of 145138 increasing from right to left). The *sn*-1/3-specific lipase should produce 1,2-DG and 2,3-TF-DG from TF-TG, whereas the non-specific lipase should produce 1,2-DG, 1,3-TF-DG, and 2,3-TF-DG (Fig. S5A). In this experiment, 1,2-DG could not be visualized on the TLC plate because it was not labeled with fluorescence. 1,3-TF-DG and 2,3-TF-DG were generated by the action of 145138 at low concentrations of the enzyme (Fig. 5A, lanes 6–12). The molar ratio of 1,3-TF-DG and 2,3-TF-DG generated by 145138 was approximately 2:1 (Fig. 5B), suggesting that 145138 hydrolyzed each acyl-ester linkage with the same efficiency. With an increase in 145138, 1,3-TF-DG and 2,3-TF-DG disappeared and TF-MG and TF-FFA were generated, indicating that TF-DGs were converted by 145138 to TF-FFA via TF-MG (Fig. 5A, lanes 2–6). These results indicated that 145138 is a non-specific lipase with no positional specificity toward TG. In addition to non-specific TG lipase activity, 145138 seems to catalyze a transesterification reaction because TF-ethyl ester (TF-EE) was generated as a byproduct (Fig. 5A, Fig. S5A) when 25% ethanol was added to the reaction mixture. Ethanol was added to the reaction because ethanol enhanced the activity of 145138 (Fig. S3D). MS assigned TF-EE as *m/z* 441.3 corresponding to the fluorine desorbed positive ion [M + H-HF]^+^ (Fig. S5C). Authentic TF-TG was also assigned as *m/z* 1015.9 corresponding to [M + H-HF]^+^ (Fig. S5B), as described in^26^. These results indicated that 145138 transferred TF-FA from the TF-TG to ethanol (Fig. S5A). The TF-EE generated was hydrolyzed when more than 12.5 ng of 145138 was used for the reaction (Fig. 5A, lane 1).

### Positional specificity (regiospecificity) of 145138 to phospholipids

As shown in Fig. 4A, 145138 completely hydrolyzed TF-TG to TF-FFA, whereas this lipase hydrolyzed NBD-PC to NBD-LPC, but NBD-FFA was barely generated. The generation of NBD-LPC increased with increasing amounts of 145138; however, almost no NBD-FFA was produced even if the enzyme amount was increased (Fig. 5C), indicating that 145138 specifically hydrolyzed the *sn*-1 acyl ester linkage of NBD-PC (Fig. S6A). Subsequently, we used PC38:6, composed of palmitic acid (16:0) at *sn*-1 and DHA (22:6) at *sn*-2, as a substrate for 145138 (Fig. S6B). As a result, LPC22:6 was generated, but almost no LPC16:0 was generated by 145138 (Fig. 5D, S6C,D), indicating that the enzyme released 16:0 at *sn*-1 from PC38:6 (Fig. S6B). To eliminate the possibility that 145138 did not release DHA from PC, we examined the hydrolysis of PC32:0, PC38:6, and PC44:12 by 145138. As a result, LPC16:0 was released from PC32:0 but not from PC38:6, in which the 16:0 is linked to PC at *sn*-1 (Fig. 5D). On the other hand, LPC22:6 was generated from not only PC38:6 but also PC44:12 (Fig. 5D), indicating that DHA can be released from PC by 145138. The release of LPC16:0 was not observed from PC38:6 even after incubation with 145138 for 240 min, whereas LPC22:6 was released from PC38:6 after 60 min (Fig. 5E). Similarly, LPE16:0 was hardly released from PE38:6, whereas LPE22:6 was efficiently generated from PE38:6 (Fig. 5F). These results clearly indicated that 145138 specifically hydrolyzes the acyl ester linkage at *sn*-1 in PC and PE, whereas acyl ester linkage at *sn*-2 is resistant to hydrolysis by 145138. In other words, 145138 possesses phospholipase A_1_ activity but not A_2_ or B activity toward PC and PE. On the other hand, NBD-phosphatidic acid (PA), which has NBD-FA with *sn*-2 position (Fig. S4), was hydrolyzed by 145138 to generate not only NBD-LPA but also NBD-FFA (Fig. S6E,F), indicating that 145138 shows both phospholipase A_1_ and A_2_ activity toward PA; however, the hydrolysis efficiency was considerably lower than that toward NBD-PC (Fig. S6G). We further confirmed that 145138 does not show phospholipase C activity as *p*NP was not released from *p*NP-phosphate by 145138 (Fig. S6H). Collectively, the positional specificity of 145138 depends on the head group of glycerolipids, i.e., 145138 hydrolyzes the acyl-ester linkage at *sn*-1 of glycerophospholipids that contain alcohol such as choline or ethanolamine linked to the phosphate group, while the enzyme nonspecifically hydrolyzes acyl-ester linkage with no-head group (TG) or non-alcoholic phosphate (PA). To the best of our knowledge, such positional specificity is unique among lipases/phospholipases reported so far.

### Determination of catalytic amino acid residue of 145138

**Figure 6:**
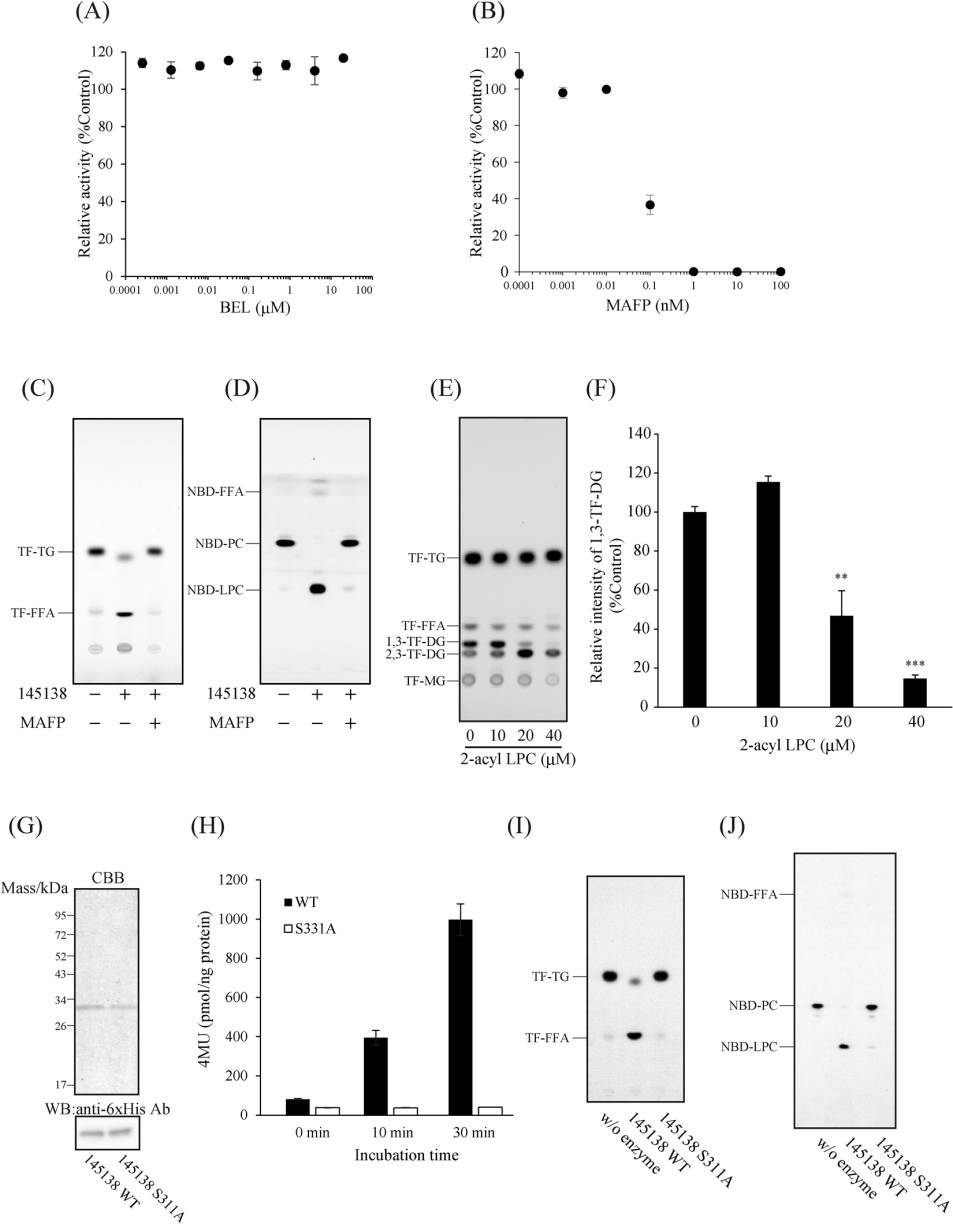
Identification of the catalytic amino acid residue of 145138. (**A**) Effect of bromoenol lactone (BEL) and (**B**) methoxy arachidonyl fluorophosphonate (MAFP) on the activity of 145138. 145138 was pre-incubated with different concentrations of BEL and MAFP, then the lipase activity was measured using 4MU-16:0 as a substrate. TLC analysis showing TG lipase activity toward TF-TG (**C**) and phospholipase activity toward NBD-PC (**D**) of 145138 with (+) or without (−) MAFP. (**E**) Competitive inhibition of TG lipase activity of 145138 by 2-acyl LPC. Different concentrations of 2-acyl LPC were added to the reaction mixture containing TF-TG and 145138. (**F**) Quantification of 1,3-TF-DG generated from TF-TG by 145138. Fluorescence intensity of 1,3-TF-DG was quantified using a fluorescence chromatoscanner. (**G**) Purification of 145138 and 145138 S311A mutant. Each of 145138 and S311A mutant was expressed in *A. limacinum* as 6× His-tagged protein, and they were purified as described in the supplemental methods. Each purified protein was subjected to SDS-PAGE and western blotting analysis with an antibody against the 6× His tag. (**H**) Lipase activities of 145138 WT and 145138 (S311A) mutant. Lipase activities were measured using 4MU-palmitic acid as a substrate. Error bars represent means ± S.D. of three separate experiments. TG lipase activity toward TF-TG (**I**) and phospholipase activity toward NBD-PC (**J**) of 145138 WT and 145138 (S311A) mutant. One hundred pmol of each substrate was incubated without (w/o) or with 25 ng of 145138 WT or 145138 (S311A) mutant at 37 °C for 30 min. TLC plates were developed with hexane/diethyl ether/acetic acid (50/50/1, v/v/v) for TF-TG and chloroform/methanol/water (65/35/8, v/v/v) for NBD-PC.

Bromoenol lactone (BEL) and methoxy arachidonyl fluorophosphonate (MAFP) are commonly used as an irreversible inhibitors of Ca^2+^-independent PLA_2_ (iPLA_2_) possessing a GXSXG lipase consensus motif^27,28^. Although BEL barely inhibited 145138 (Fig. 6A), we found that MAFP strongly inhibited 145138 at low concentration (Fig. 6B). The activity of 145138 was completely inhibited by MAFP when either TF-TG or NBD-PC was used as a substrate (Fig. 6C,D). Furthermore, 2-acyl LPC competitively inhibited the release of fatty acyl chain at *sn*-2 of TG (Fig. 6E,F). These results suggest that the catalytic site of 145138 is the same for TG and glycerophospholipids. To determine the catalytic amino acid in 145138, the serine residue in GXSXG, serine 331 (S331), was replaced with alanine (A) then expressed as 145138 S311A in *A. limacinum* (Fig. 6G). After purification, the lipase activities of 145138 WT and 145138 S311A with 4MU-palmitate, TF-TG, and NBD-PC were measured. 4MU-palmitate was degraded by 145138WT, but not by the 331 A mutant (Fig. 6H). Moreover, lipase activity toward TF-TG and phospholipase A_1_ activity toward NBD-PC were significantly decreased in the S311A mutant (Fig. 6I,J). These results indicated that S311 in GXSXG is a nucleophilic serine similar to conventional lipases, and that the same catalytic residue is used for the lipase and phospholipase activities of 145138, even though the positional specificities are different.

### Requirement of 145138 for assimilation of TG in A. limacinum

**Figure 7:**
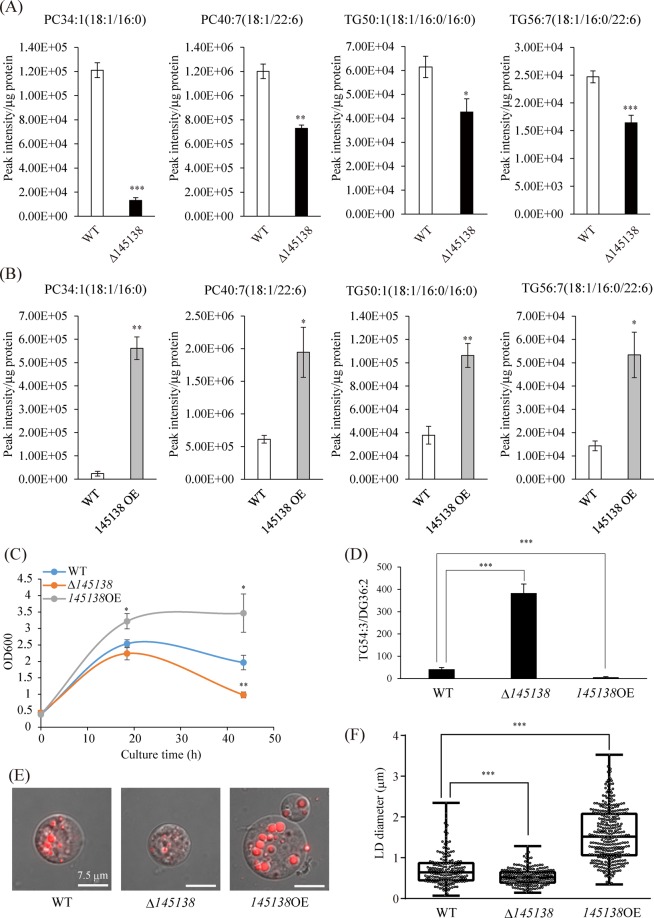
Contribution of 145138 to the assimilation of TG in *A. limacinum*. (**A**) Incorporation of triolein TG54:3 (18:1/18:1/18:1)-derived oleic acid into different molecular species of PC and TG in Δ145138. TG54:3 (18:1/18:1/18:1) was added to the culture of *A. limacinum* to reach 400 μM, and cells were collected by centrifugation after a 24-hour incubation. Total lipids were extracted from cells and subjected to LC-ESI MS/MS. (**B**) The same experiment as (**A**) was performed using 145138OE instead of Δ*145138*. Cells were collected after a 1-hour incubation with 400 μM TG54:3 in this experiment. (**C**) Growth curves for WT (blue), Δ145138 (orange), and 145138OE (grey) in medium in which the only carbon source is TG54:3 (TG-medium). (**D**) The ratio of remaining TG54:3 and DG36:2, generated from TG54:3, in the medium of WT, Δ145138, and 145138OE. Error bars represent means ± S.D. of three separate experiments. (**E**) LDs of WT, Δ145138 and 145138OE strains cultured in TG-medium. LDs were stained with HCS LipidTOX Red neutral lipid stain. Scale bar represent 7.5 μm. (**F**) Diameter of LDs in WT, Δ145138, and 145138OE. Diameters were measured using LAS X software.

We found that TG was present in the culture medium of *A. limacinum* after the stationary phase, possibly due to leakage from dead cells. TG levels decreased in the medium of the 145138OE line with an increase in lipase activity in the medium, suggesting that 145138 is involved in the hydrolysis of extracellular TG for utilization as a lipid-nutrient (Fig. 3A). Because *A. limacinum* hardly synthesizes oleic acid (18:1)^29^, 18:1 could be used as a tracer for the biosynthesis of PC and TG, which would provide evidence of lipid assimilation of extracellular fatty acids generated by 145138. In this study, we added TG54:3 (triolein, 18:1/18:1/18:1) to the medium of WT, Δ145138, and 145138OE strains of *A. limacinum*, and thereafter the amounts of cellular 18:1-containing PC and TG were examined by mass spectrometry. As expected, the incorporation of 18:1 into cellular PC and TG decreased in Δ145138, whereas it increased in the 145138OE line (Fig. 7A,B), indicating that 18:1 released from TG54:3 was incorporated into *A. limacinum* cells after hydrolysis by 145138 and used for the synthesis of 18:1-containing TG and PC. To clarify the biological importance of 145138-mediated lipolysis, WT, Δ145138, and 145138OE strains were cultured in medium containing TG54:3 as the sole carbon source (TG-medium). As mentioned above, the growth curve were not changed by the disruption or overexpression of 145138 when cultured in GY medium, in which carbon source is glucose (Figs S1C, S2B). In contrast, growth suppression occurred in Δ145138 and improvement of growth was observed in 145138OE in TG-medium (Fig. 7C). In agreement with this result, the amount of remaining TG in the medium was increased in Δ145138 and decreased in 145138OE (Fig. 7D). Moreover, we found that the development of LDs was suppressed in Δ145138 and enhanced in 1454138OE (Fig. 7E,F). On the other hand, no difference in LD size was observed among strains when cultured in GY medium (Fig. S7A,B). The growth of all the strains was almost the same in the medium containing free 18:1 as the sole carbon source (Fig. S7C), suggesting that the difference in the growth in the TG-medium depends on the availability of TG54:3 by 145138. These results indicated that 145138 is a major secretory lipase that is indispensable for the utilization of extracellular TG as a lipid-nutrient for the survival of thraustochytrids.

### Antimicrobial activity of LPC generated by phospholipase A_1_ activity of 145138

**Figure 8:**
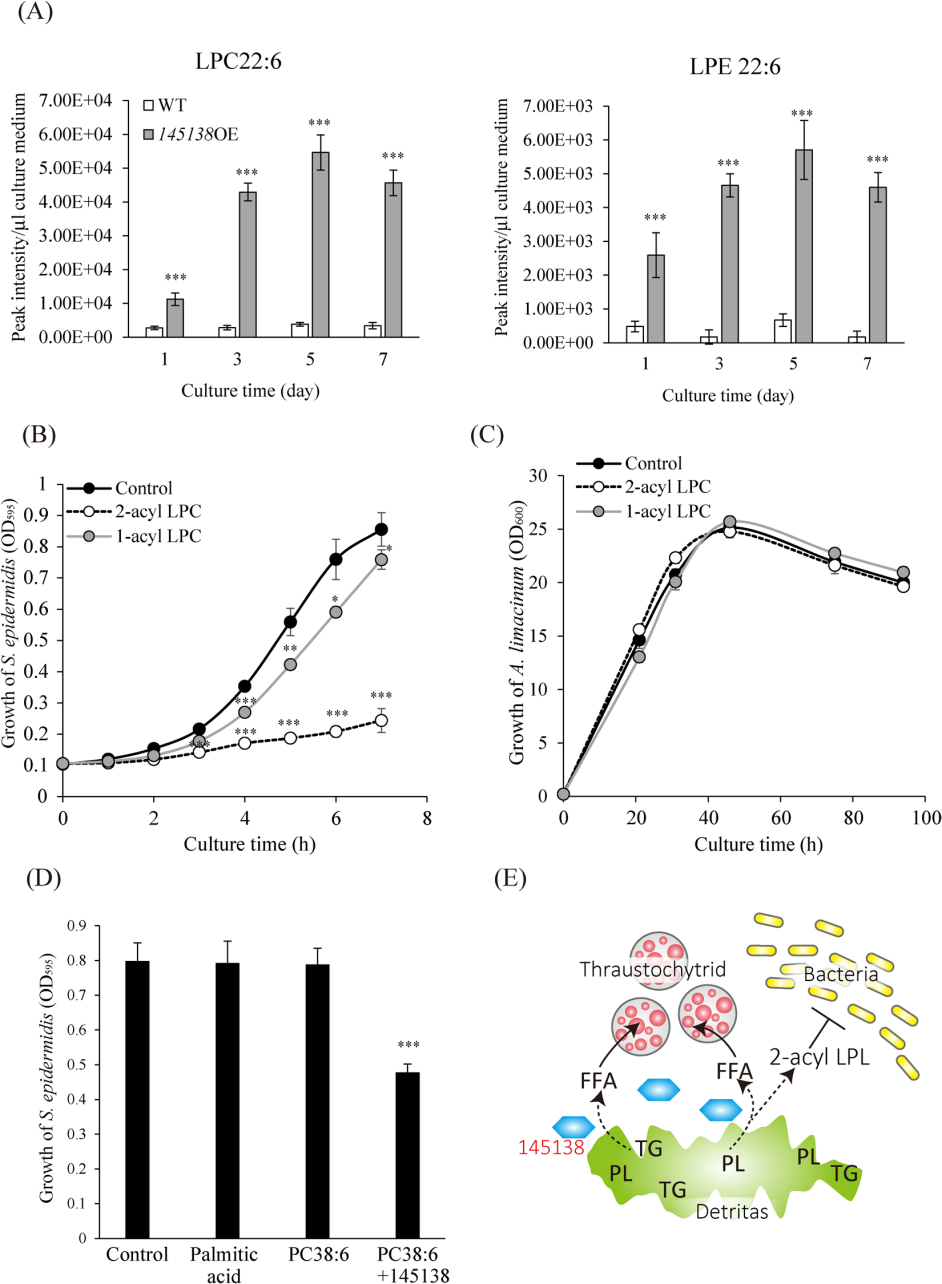
Antimicrobial effect of LPC generated by phospholipase A_1_ activity of 145138. (**A**) The amounts of extracellular LPC22:6 and LPE22:6 in WT and 145138OE. LPC22:6 and LPE22:6 in the culture supernatants were measured using LC-ESI MS/MS at the time points indicated. (**B**) Antimicrobial activity of 1-stearoyl-2-hydroxy-sn-glycero-3-phosphocholine (1-acyl LPC) and 2-stearoyl-sn-glycero-3-phosphocholine (2-acyl LPC) on gram-positive bacteria (*S. epidermidis*). Six nmol of 1-acyl LPC or 2-acyl LPC dissolved in ethanol was added to 150 μl of culture medium of *S. epidermidis*, respectively. (**C**) Effect of 1-acyl and 2-acyl LPC on the growth of *A. limacinum*. Sixty nmol of 1-acyl LPC or 2-acyl LPC dissolved in ethanol was added to 1.5 ml of culture medium of *A. limacinum*, respectively (**D**) Antimicrobial effect of palmitic acid and LPC released from PC38:6 by 145138. PC38:6 was digested by 145138 then almost all PC38:6 was converted to LPC22:6 (Fig. S8C). Six nmol of each compound was added to the culture medium (150 μl) of *S. epidermidis*. The growth of *S. epidermidis* was monitored by measuring OD_595_ using a plate reader. Ethanol was used as a control for the antimicrobial activity of LPC. (**E**) Estimated role of 145138 for thraustochytrids. 145138 is the major extracellular lipase that degrades TG derived from marine detritus to generate FFAs, which can be incorporated into thraustochytrid cells and utilized as an energy source or building block for phospholipids or TG synthesis. 145138 can release all fatty acids in TG, while it hydrolyzes the ester bond in the *sn*-1 position in phospholipids selectively, generating 2-acyl lysophospholipid (2-acyl LPL) and FFA. Antimicrobial activity of 2-acyl LPL enables thraustochytrids to obtain nutrients preferentially in the marine environment even in the presence of bacteria. The novel secretory lipase/phospholipase 145138 is beneficial for thraustochytrids to assimilate lipids from the marine environment, where thraustochytrids are surrounded by numerous competitors such as bacteria.

Consistent with a decrease in PC38:6 and PE38:6 (Fig. 3B), LPC22:6 and LPE22:6 significantly increased in the medium of the 145138OE line (Fig. 8A). DHA is mainly linked to *sn*-2 of PC and PE in *A. limacinum*^30^, indicating that secreted 145138 shows phospholipase A_1_ activity and generates lysophospholipids into the medium in practice. If 145138 is just used for the degradation and assimilation of lipid nutrients, it is more advantageous for thraustochytrids to let 145138 have phospholipase B activity, then all fatty acids in phospholipids can be incorporated into cells. It should be noted that lysophospholipids have antimicrobial activity^31^. Thus, we hypothesized that 145138-derived lysophospholipid may act as an antibiotic, which would be advantageous for thraustochytrids in microbial competition. Notably, we found that 2-acyl LPC, which is the product of phospholipase A_1_, showed higher antimicrobial activity than 1-acyl LPC toward gram-positive bacteria *Staphylococcus epidermidis* (Fig. 8B, Fig. S8A), and gram-negative bacteria *Pseudomonas aeruginosa* (Fig. S8B). Since fatty acyl chain at *sn*-2 in 2-acyl LPC could move to the *sn*-1 position generating 1-acyl LPC by acyl-migration^32^, actual 2-acyl LPC level in the medium was carefully examined by the LC-MS/MS method^32^, which can separate and quantify 1-acyl and 2-acyl LPC. Importantly, 2-acyl LPC still remained in the medium at sufficient level even though acyl migration had slightly occurred in this experimental condition (Fig. S9), indicating that effective antimicrobial activity observed in 2-acyl LPC (Fig. 8B) was certainly caused by 2-acyl LPC added into the medium. On the other hand, 1-acyl and 2-acyl LPC did not affect the growth of *A. limacinum* (Fig. 8C). These results suggested that the unique positional specificity of 145138 may contribute to generate antibacterial compounds in their environment. In agreement with a previous report^31^, LPC showed more effective antimicrobial activity against gram-positive bacteria. Thus, we focused on gram-positive bacteria in this study. As expected, LPC22:6 generated from PC38:6 by 145138 (Fig. S8C) showed significant antimicrobial activity (Fig. 8D). In contrast, no antimicrobial effect was detected with palmitic acid, which is also generated from PC38:6 by 145138, or PC38:6 itself (Fig. 8D). As group IIA secretory phospholipase A_2_ (sPLA_2_-IIA) shows direct antimicrobial activity^33^, we tested whether 145138 directly affects the growth of gram-positive bacteria. No antimicrobial activity was observed when bacteria were cultured in the medium containing145138 (Fig. S8D), indicating that this enzyme is unable to directly affect the bacterial growth. Taken together, 145138 is considered as a bifunctional enzyme that is used not only for the degradation of environmental TG for assimilation but also for generating antibiotics to attack bacteria, which are major decomposers in the marine environment, providing a strong competitive advantage for thraustochytrids (Fig. 8E).

## Discussion

In this study, we identified novel thraustochytrid-specific lipase-like genes by comparative genomic clustering analysis in Stramenopiles. It should be noted that these novel genes were not obtained by the general homology searches because of the low sequence similarities with known secretory lipases. Only 145138 and 33542, when expressed in *E. coli*, showed lipase activity toward TG, whereas 150216, 149169, 5590, and 2999 did not (Fig. 1D). One possibility is that these lipase-like gene products have no lipase activity, as seen for pancreatic lipase-related protein 1 (PLRP1)^34^. PLRP1, a homologue of pancreatic lipase, does not hydrolyze TG or phospholipids, rather PLRP1 inhibits pancreatic lipase activity through binding to lipase activator^35^, through which PLRP1 regulates lipolysis *in vivo*^34^. Actual functions of lipase-like genes other than 145138 are expected to be clarified by expressing and/or deleting them in *A. limacinum* as we did for 145138 in this study. Because extracellular lipase activity and fatty acid assimilation from TG were dramatically decreased in Δ145138 (Fig. 2B, Fig. 7A,C), we emphasize that 145138 is considered as the major secretory lipase involved in lipid degradation for *A. limacinum* among thraustochytrid-specific lipase-like genes.

Of note, although 145138 was an entirely secreted protein, no known secretory signal sequence was found in the N- or C- terminal regions of 145138. On the other hand, we found that 159 amino acid residues at the N-terminal of 145138 are cleaved off during the secretary process (Fig. S2C). Thus, these N-terminal amino acid residues (Fig. S2E) would act as a signal sequence for the secretion of 145138. As the N-terminal region of pre-secretory form of 145138 is not similar to any other known proteins, a novel system for protein secretion is probably present in *A. limacinum*. Further studies are needed to understand the mechanism of the novel secretion system.

145138 is unique in its positional specificity as well as its primary structure, as described in this study. Based on its properties, 145138 can be designated as thraustochytrid-specific secretion lipase/phospholipase A_1_ (accession number: LC379240). Notably, the 2-acyl LPC that is derived from PC by the phospholipase A_1_ activity of 145138 showed significant antimicrobial activity, indicating that the unique positional specificity of 145138 may contribute to produce antibiotics that are beneficial for competition with bacteria. Marine bacteria also assimilate exogenous fatty acids^36^, thus fatty acids generated by 145138 from TG, which might be derived from detritus, would become generally available nutrients that could be intercepted by such bacteria in marine environments in the absence of antibiotic production (Fig. 8E). The possibility that extracellular lysophospholipids act as a signal molecule in cell–cell communications or chemotaxis cannot be ruled out because lysophospholipids are also known to act as inter- and intracellular signaling molecules^37^. At present, how 145138 shows different positional specificities for TG and phospholipids is unclear at the molecular level. Importantly, we found that 145138 could release not only *sn*-1, but also *sn*-2 fatty acyl chains from PA wherein the head group lacks the alcohol moiety linked to the phosphate group. Thus, it is plausible that the accessibility of 145138 toward the *sn*-2 acyl-ester linkage of phospholipids could be inhibited by the alcohol moieties such as choline or ethanolamine in the head group at *sn*-3. Because no homology was found between 145318 and lipases reported so far except for the lipase motif (GXSXG) (Fig. 1B), it is impossible to perform homology modeling of 145138 using known lipases as a template. Thus, crystal structural analysis is needed to understand the molecular mechanism underlying the unique positional specificity of 145138.

Our result also indicated the possible usefulness of 145138 for industrial uses. Organic solvent tolerance is an important factor for lipases for industrial applications because the high dissolution of hydrophobic lipids, minimization of water-dependent unwanted reactions, and a shift from hydrolysis to synthesizing reactions such as transesterification for producing biodiesel or fine chemicals^38,39^. Because 145138 shows high efficiency for transesterification to ethanol with high tolerance for organic solvents, this lipase could be useful for biodiesel production. Lipases are widely used in fermentation and ripening steps in the food industry. Because a high concentration of salt is used in these steps, lipases used in food elaboration need to be tolerant to salt^40^. Thraustochytrids originally come from a marine environment, and 145138 is a secreted lipase; therefore, this lipase may work in high salt environments in nature. Thus, it is reasonable that 145138 is highly resistant to NaCl, which makes it a suitable lipase for use in the food industry. Furthermore, 145138 was able to hydrolyze the DHA-ester linkage of TG and PC (Figs 4D, 5D). This nature is unique, because the DHA-ester linkage is somewhat resistant to hydrolysis by other known lipases^41^. Collectively, 145138 could become a suitable lipase for industrial use because of its high tolerance to organic solvents and salts, and broad substrate specificity toward fatty acyl chains of neutral lipids and phospholipids.

Homologs of 145138 were exclusively found in thraustochytrids in Stramenopiles indicating that thraustochytrids have acquired unique lipase(s) through their own evolutionary process. In this study, 145138 is revealed as the major extracellular lipase that is indispensable for the assimilation of extracellular lipids for *A. limacinum*. As shown in Figs 2 and 3, considerable amounts of TG and phospholipids composed of DHA were detected in the medium. These extracellular lipids might be derived from LDs released from dead cells of *A. limacinum*. DHA is an integral fatty acid for thraustochytrids^42^, thus recycling of DHA between dead and living cells may occur in a group of thraustochytrids. FFAs including DHA released from TG or phospholipids by 145138 could be transported into cells via fatty acid transporters, of which candidate genes are detected in the draft genome of thraustochytrids.

Our results strongly suggested that thraustochytrids may act as a lipid-decomposer and contribute to the circulation of lipid-derived organic matter in the marine ecosystem.

## Methods

### Strains and culture

*A. limacinum* mh0186, isolated from seawater of the Yaeyama Islands in Okinawa, Japan^43^, was grown in GY medium (3% glucose and 1% yeast extract in 1.75% artificial sea water) at 25 °C with rotation at 150 rpm for the period indicated. Potato dextrose agar (PDA) plates (50% potato dextrose, 1.75% artificial sea water, 2% agar) containing appropriate antibiotics were used to select the 145138OE and Δ145138 lines. The growth (biomass) of *A. limacinum* was monitored by measuring optical density at 600 nm (OD 600). Triolein was added to Y medium (1% yeast extract in 1.75% arficial sea water) at a final concentration of 5 mM to prepare the TG-medium.

### Searching for the thraustochytrid-specific lipase genes

The Joint Genome Institute provides a database appropriate for gene clustering including in Stramenopiles including thraustochytrids. Gene clustering was performed by the TRIBE-MCL clustering method^44^ from all- vs all- BLAST of the proteins in Stramenopiles. As a result, putative genes in cluster number 3237, 7307, and 14632 containing a lipase class 3 motif (PF01764) were selected as thraustochytrid-specific lipase-like genes. In this study, protein IDs: 2999, 5590, 33542, 145138, 149169, and 150216 were selected as lipase-like gene products of *A. limacinum*. The amino acid sequences of these lipase-like genes were aligned with those of known lipase class 3 proteins using MUSCLE^45^, and a phylogenic tree was constructed by the neighbor-joining method using MEGA7^46,47^. The robustness of the tree was evaluated using the bootstrap method (1000 repeats).

### LC-ESI MS/MS analysis

Seventy-five μl of culture fluid was collected from WT, Δ145138, and 145138OE every 2 days. The cell pellet and supernatant were separated by centrifugation at 5,000 × g for 3 min and used as cellular and extracellular fractions, respectively. Forty μl of supernatant was transferred to a new 1.5 ml tube and mixed with 160 μl of 2-propanol, then centrifuged at 17,800 × g for 5 min to remove insoluble compounds. One hundred eighty μl of the supernatant was transferred to autoinjector vials, which were used for measuring the content of extracellular PC, PE, LPC, LPE, and TG. The cell pellet was dissolved in 150 μl distilled water, then crushed at 3,000 rpm for 60 sec using a bead beater (μT-12, TAITEC) with glass beads (diameter: 0.6 mm, AS ONE Corp.), and kept on ice for 60 sec. This procedure was repeated 3 times to prepare the cell lysate. Cellular lipids were extracted from 40 μl of cell lysate by adding 160 μl of chloroform/methanol (2:1, v/v) containing 10 μM PC22:0 (11:0/11:0), 20 μM PE24:0 (12:0/12:0), 10 μM LPC13:0, 20 μM LPE13:0, and 20 μM TG36:0 (12:0/12:0/12:0) as internal standards. After incubation at 37 °C for 30 min at 150 rpm, the mixture was centrifuged at 11,000 × g for 3 min. Forty microliters of the organic phase was transferred to autoinjector vials containing 560 μl of 2-propanol, and then the cellular lipids were measured using LC-ESI MS/MS (3200 QTRAP, SCIEX). A binary solvent gradient with a flow rate of 200 μl/min was used to separate phospholipids and neutral lipids by reverse-phase chromatography using an InertSustain C18 column (2.1 × 150 mm, 5 μm, GL Sciences) as described in^9,48^. LPC, LPE, PC, PE, and TG containing palmitic acid, docosapentaenoic acid (22:5n-6), and DHA were detected using a multiple reaction monitoring (MRM) as described in^9^. FFAs were separated by reverse-phase chromatography using InertSustain C18, using a gradient starting with 10% solvent B2 (methanol with 2.5 mM ammonium acetate) in solvent A2 (distilled water with 2.5 mM ammonium acetate) and reached 90% solvent B2 for 1 min, then 95% solvent B2 for 15 min. The column was equilibrated for 5 min before the next run. MRM conditions in negative ion mode were as follows, DHA (Q1/Q3 = 327.3/327.3), palmitic acid (Q1/Q3 = 255.1/255.1).

### Measurement of lipase activity using 4MU-palmitate

Lipase activities in the culture supernatants of the WT, Δ145138, and 145138OE lines were measured using 4MU-palmitate (Sigma-Aldrich) as a substrate^20^. Briefly, 10 μl of the culture supernatant was mixed with 180 μl of 50 mM Tris-HCl, pH 8, containing 2 nmol of 4MU-palmitate. To assess the effect of inhibitors, BEL or MAFP, 145138 was pre-incubated with several concentrations of BEL for 10 min, or MAFP for 5 min, then mixed with 4MU-palmitate. The reaction mixture was kept at 37 °C for the appropriate time, and 4MU released was measured using a Wallac 1420 ARVO fluorescence microplate reader set at 355 nm Ex/460 nm Em (PerkinElmer).

### Substrate specificity of 145138

An aliquot of 100 pmol of TF-TG, TF-DG, TF-MG, BODIPY-CE, NBD-PC, NBD-PA, NBD-ceramide, or NBD-GlcCer was incubated with 50 ng of 145138 in 100 μl of 50 mM Tris-HCl buffer, pH 8, containing 20 mM of CaCl_2_ and 25% ethanol at 37 °C for an appropriate period with 2000 rpm rotation. The reaction was stopped by adding 400 μl of CHCl_3_/MeOH (2/1, v/v), and the sample was centrifuged (11,000 × g for 3 min). The organic phase was dried using a speed vac concentrator, dissolved in 30 μl chloroform/methanol (2/1, v/v), and then 5 μl was applied to a TLC plate, which was developed with hexane/diethyl ether/acetic acid (50/50/1, v/v/v) for TF-TG, TF-DG, TF-MG and BODIPY-CE, chloroform/methanol/water (65/35/8, v/v/v) for NBD-PC, NBD-PA, NBD-ceramide, and NBD-GlcCer, respectively. Substrate specificity for phospholipid head group of 145138 was measured by using 1 nmol of PC32:0 and PE32:0, respectively. Five ng of 145138 in 100 μl of 50 mM Tris-HCl buffer, pH 8, containing 20 mM of CaCl_2_ and 25% ethanol was incubated with PC32:0 or PE32:0 at 37 °C for 20 min with rotation at 2000 rpm. Kinetic constants of 145138 were measured by using TG 48:0 (16:0/16:0/16:0) and TG 66:18 (22:6/22:6/22:6) at concentrations ranging from 0.01 to 100 μM. For this reaction, reaction mixture (100 μl of 50 mM Tris-HCl buffer, pH 8, containing 20 mM of CaCl_2_ and 25% ethanol) was kept at 37 °C for 60 min with rotation at 2000 rpm. The reaction was stopped by adding 400 μl of chloroform/methanol (2/1, v/v), then the mixture was centrifuged (11,000 × g for 3 min), and 120 μl of the organic phase was mixed with 480 μl 2-propanol, then transferred to autoinjector vials. Lipase activity was calculated from the amount of remaining TG, PC, or PE by using LC-ESI MS/MS. Kinetic parameters were calculated by nonlinear regression using GraphPad Prism6 (GraphPad Software Inc.).

### Positional specificities of 145138 for TG and PC

An aliquot of 100 pmol of TF-TG or NBD-PC was incubated at 37 °C for 20 min with rotation at 2000 rpm with different amounts of 145138 (6 pg to 12.5 ng for TF-TG, 50 pg to 12.5 ng for NBD-PC) in 100 μl of 50 mM Tris-HCl buffer, pH 8, containing 20 mM of CaCl_2_ and 25% ethanol. Enzymatic products were measured using TLC, as described above. An aliquot of 1 nmol of PC32:0 (16:0/16:0), PC38:6 (16:0/22:6), PC44:12 (22:6/22:6), and PE38:6 (16:0/22:6) was incubated at 37 °C for an appropriate time with rotation at 2000 rpm with 5 ng of 145138 in 100 μl of 50 mM Tris-HCl buffer, pH 8, containing 20 mM of CaCl_2_ and 25% ethanol. LPC or LPE were detected by Q1 scanning and quantified by MRM analysis using LC-ESI MS/MS^9^.

### Incorporation of triolein-derived fatty acid into A. limacinum

WT, Δ145138, and 145138OE were cultured at 25 °C in a 50 ml GY medium for 5 days, then triolein was added to each culture at a final concentration of 400 μM. Cells were collected from each culture after adding triolein for 1 and 24 h. Lipids were extracted from cell lysates as described above. Oleic acid-containing PC and TG were measured using MRM with LC-ESI MS/MS.

### Staining and observation of LDs

LDs were stained by HCS LipidTOX Red neutral lipid stain (Thermo Fisher Scientifc), and were observed under the fluorescence microscope DMi8 with Leica Application Suite X (LAS X) equipped with an objective lens of ×100 (numerical aperture 1.40) and a DFC3000G camera (Leica Microsystems).

### Antimicrobial activity of LPC

*S. epidermidis* and *P. aeruginosa* were incubated at 37 °C with shaking in LB medium that contains appropriate amount of LPC dissolved in ethanol. The growth of *S. epidermidis* and *P. aeruginosa* were monitored by measuring OD_595_ by plate reader Multiskan FC (Thermo Fisher Scientific). PC38:6 was incubated with 25 ng of 145138 for 120 min at 37 °C in 100 μl of 50 mM Tris-HCl buffer, pH 8, containing 20 mM of CaCl_2_ and 25% ethanol. After incubation, 400 μl of chloroform/methanol (2:1, v/v) was added to the reaction mixture, centrifuged at 11,000 × g for 3 min, then the organic phase was evaporated by speed vac concentrator. LPC22:6 generated from PC38:6 was dissolved in ethanol with sonication, then added to the 150 μl of the LB medium. Ethanol was used as a control for the antimicrobial activity of LPC. To test the direct antimicrobial activity of 145138, the purified 145138 was added to the LB medium, which contains 50 mM Tris-HCl buffer (pH 8) and 20 mM CaCl_2_, at the concentration of 100 ng/ml.

### Statistical analysis

All statistical analyses were performed using unpaired two-tailed Student’s t-tests, and all data are expressed as means and standard deviation from at least three separate experiments. Statistical significance is indicated as follows: *p < 0.05; **p < 0.01; ***p < 0.001.

## Supplementary information

Supplementary information
