# Supplementary material for: A thraustochytrid-specific lipase/phospholipase with unique positional specificity contributes to microbial competition and fatty acid acquisition from the environment
Source: Sci Rep. 2019 Nov 8;9:16357. doi: 10.1038/s41598-019-52854-7 (PMC6841712; doi:10.1038/s41598-019-52854-7)
Supplement: Supplementary file 1 — Supplementary information [file 41598_2019_52854_MOESM1_ESM.docx]

**Supplementary information**

**A thraustochytrid-specific lipase/phospholipase with unique positional specificity contributes to microbial competition and fatty acid acquisition from the environment**

Yohei Ishibashi^1,*^, Keisuke Aoki^1^, Nozomu Okino^1^, Masahiro Hayashi^2^, Makoto Ito^1,3^

**Supplemental Methods**

*Materials* – 1,2-diundecanoyl-*sn*-glycero-3-phosphocholine [PC22:0 (11:0/11:0)], 1,2-dipalmitoyl-*sn*-glycero-3-phosphocholine [PC32:0 (16:0/16:0)], 1,2-dilauroyl-sn-glycero-3-phosphoethanolamine [PE24:0 (12:0/12:0)], 1,2-dipalmitoyl-*sn*-glycero-3-phosphoethanolamine [PE32:0 (16:0/16:0)], 1-palmitoyl-2-docosahexaenoyl-*sn*-glycero-3-phosphocholine [PC38:6 (16:0/22:6)], 1-palmitoyl-2-docosahexaenoyl-*sn*-glycero-3-phosphoethanolamine [PE38:6 (16:0/22:6)], 1,2-didocosahexaenoyl-*sn*-glycero-3-phosphocholine [PC44:12 (22:6/22:6)], 1-tridecanoyl-2-hydroxy-*sn*-glycero-3-phosphocholine (LPC13:0), 1-stearoyl-2-hydroxy-sn-glycero-3-phosphocholine (1-acyl LPC), 2-stearoyl-*sn*-glycero-3-phosphocholine (2-acyl LPC), 1-tridecanoyl-*sn*-glycero-3-phosphoethanolamine (LPE13:0), 1-oleoyl-2-[12-[(7-nitro-2-1,3-benzoxadiazol-4-yl)amino]dodecanoyl]-*sn*-Glycero-3-PC (NBD-PC), 1-oleoyl-2-[12-[(7-nitro-2-1,3-benzoxadiazol-4-yl)amino]dodecanoyl]-*sn*-glycero-3-phosphate (NBD-PA), 1,2-dioleoyl-3-[11-(dipyrrometheneboron difluoride)undecanoyl]-*sn*-glycerol (TF-TG), 1-oleoyl-3-[11-(dipyrrometheneboron difluoride)undecanoyl]-rac-glycerol (TF-DG), and 1-[11-(dipyrrometheneboron difluoride)undecanoyl]-rac-glycerol (TF-MG) were purchased from Avanti Polar Lipids. TG36:0(12:0/12:0/12:0) (Trilaurin), TG48:0(16:0/16:0/16:0) (Tripalmitin), and TG54:3(18:1/18:1/18:1) (Triolein) were purchased from Tokyo Chemical Industry. TG66:18(22:6/22:6/22:6) (Tridocosahexaenoin), methyl arachidonyl fluorophosphonate (MAFP), and (S)-bromoenol lactone (BEL) were purchased from Cayman Chemical. Antibodies against anti-His-tag (#2366), anti DYKDDDDK tag (#2368), anti-rabbit IgG horseradish peroxidase (HRP), and anti-mouse IgG HRP were obtained from Cell Signaling Technology. TLC and p-nitrophenyl phosphate were purchased from Merck Millipore. Cholesteryl 4,4-difluoro-5,7-dimethyl-4-bora-3a,4a-siaza-s-indacene-3-dodecanoate (BODIPY-CE) and 6-((N-(7-nitrobenz-2-oxa-1,3-diazol-4-yl)amino)hexanoyl)sphingosine (NBD-ceramide) were purchased from Thermo Fisher Scientific Inc. N-hexanoyl-NBD-glucosylceramide (NBD-GlcCer) was obtained from Matreya. SEALIFE, which was used as artificial seawater, was purchased from Nihonkaisui Co., Japan.

*Expression of A. limacinum lipase-like genes in E. coli* – Total RNA was extracted from *A. limacinum* mh0186 using Sepasol-RNA I Super G (Nacalai Tesque) and an SV Total RNA Isolation System (Promega). Complementary DNA (cDNA) was synthesized using a PrimeScript™ RT reagent Kit with a gDNA Eraser (Perfect Real Time) (Takara Bio Inc.). PCR was carried out using *A. limacinum* cDNA as a template, the primers listed in Table S1, and Tks Gflex™ DNA Polymerase (Takara Bio Inc). The amplified products were inserted into *Eco*RI-digested pCold I (Takara Bio Inc) to generate N-terminal 6×His-tagged recombinant proteins using an In-Fusion HD Cloning kit (Takara Bio Inc). Protein IDs: 2999, 145138, 33542, 150216, 149169, and 5590 were separately expressed in *E. coli* BL21 transformed with the prepared pCold vectors. Incubation at 37°C with shaking in Luria-Bertani (LB) medium supplemented with 100 μg/ml ampicillin was continued until OD600 nm reached ∼0.5, and then the culture was kept at 15°C for 30 min. Isopropyl β-D-thiogalactopyranoside was added to the culture at a final concentration of 1 mM to induce transcription. After being kept at 15°C for 24 h, the cells were harvested by centrifugation (8,000 × g for 10 min), suspended in 100 mM Tris HCl buffer, pH 7.5, and kept in a sonic bath for 90 s (30 s × 3). Cell lysates were centrifuged (17,800 × g for 15 min at 4°C), and the supernatant and sediment were recovered as soluble and insoluble fractions, respectively.

*Measurement of lipase activity of recombinant lipase-like proteins* – Five hundred pmol of [carboxyl-^14^C]-triolein (Perkin Elmer) was incubated at 25°C for 30 min with an equal amount of cell lysate of *E. coli* expressing lipase-like genes in 200 μl of 100 mM potassium phosphate buffer, pH 8, containing 400 μg of bovine serum albumin, and 5 μmol of MgCl_2_. After incubation, the enzyme reaction was stopped by adding 800 μl of CHCl_3_/MeOH (2/1, v/v), and centrifuged (11,000 × g for 3 min). The organic phase was withdrawn and dried using a stream of nitrogen, dissolved in 10 μl of chloroform/methanol (2/1, v/v), and then applied to a TLC plate, which was developed with hexane/diethyl ether/acetic acid (50/50/1, v/v/v). The radioisotope-labeled lipids were detected using a Typhoon FLA 9500 Bio-imaging analyzer (GE Healthcare).

*Western blotting analysis* – Proteins were subjected to 12.5% SDS-PAGE, followed by blotting onto a PVDF membrane using a Trans-Blot SD semi-dry transfer cell (BioRad). The membrane was blocked in Tris-buffered saline-Tween 20 (TBS-T) containing 5% bovine serum albumin, then incubated with antibodies against DYKDDDDK tag (1:5000) or 6×His tag (1:2500) for over 12 h at 4°C. The membrane was washed with TBS-T and then incubated with HRP-conjugated anti-mouse or rabbit IgG antibody (1:10000) (Cell Signaling Technology). The membrane was washed with TBS-T, and the protein was detected by chemiluminescence using Luminate Forte Western HRP substrate (Millipore) and visualized with the use of a Cooled CCD Camera System Ez-Capture II (ATTO). For detection of secreted 145138, proteins were precipitated from the culture supernatant of *145138*OE using cold acetone.

*Quantitative real-time PCR* – Real-time PCR was performed using an Mx3000P qPCR System (Agilent Technologies) with SYBR Premix Ex Taq II (Tli RNaseH Plus) (Takara-Bio) by using cDNA from WT (2 days and 7 days culture) and Δ*145138* cells (7 days culture) as templates. The numbers of copies of *145138* were determined using a plasmid containing *145138* as a standard. Oligonucleotide primer sets for real-time PCR are shown in Table S1.

*Generation of 145138-disrupted mutants (Δ145138)* – The *145138* gene was disrupted in *A. limacinum* mh0186 by homologous recombination in accordance with the method described in ([1](#_ENREF_1)). A DNA cassette composed of a 5’UTR (1197 bp), ORF (1358 bp), and 3’UTR (1171 bp) of 145138 was amplified by using genomic DNA of *A. limacinum* mh0186 as a template, primers (5UTR 145138-S and 3UTR 145138-A), and Tks Gflex™ DNA Polymerase. The amplicon was inserted into a pUC19 vector using an In-Fusion HD Cloning kit, and then inverse PCR was performed to eliminate the *145138* ORF by using a primer set (5UTR 145138-A and 3UTR 145138-S). The hygromycin-resistance gene expression cassette ([2](#_ENREF_2)) was inserted between the 5’UTR and 3’UTR sequences of *145138* instead of the ORF using an In-Fusion HD Cloning kit (Fig. S1). The knockout construct was amplified by PCR using the primer set of 5UTR 145138-S and 3UTR 145138-A, and introduced into *A. limacinum* mh0186 by electroporation with a Gene Pulser Xcell (750 V, 25 μF, 200 Ω) ([3](#_ENREF_3)). After being pulsed twice, 800 μl of fresh medium was added. Cells were cultured at 25°C for 1 day and then transferred to a PDA-agar plate containing 1 mg/ml of hygromycin B (Wako). Transformants were examined for disruption of *145138* by PCR-1 using the primer set 145138 KO outside-5UTR-S and HygR-A (Fig. S1A, Table S1). The disruption of *145138* was also confirmed by PCR-2 using the primer set 145138 KO outside-5UTR-S and 145138 KO outside-3UTR-A (Fig. S1A, Table S1).

*Generation of 145138-overexpressing mutants* – In order to prepare the construct expressing FLAG or 6×His tagged 145138, PCR was performed using cDNA as a template and the primer set 145138-S and 145138 FLAG-A or 145138-S and 145138FLAG-His-A (Table S1). Inverse PCR was performed to eliminate mCherry, which was located between the ubiquitin promoter and ubiquitin terminator of the expression vector of thraustochytrids, pEF-Neor/Ubi-mCherry using primer set A.L vector Rv and A.L vector Fw ([4](#_ENREF_4)) (Table S1). C-terminal FLAG or 6×His tagged 145138 was inserted between the ubiquitin promoter and ubiquitin terminator of pEF-Neor/Ubi-mCherry instead of mCherry using an In-Fusion HD cloning kit (Fig. S2). The plasmid construct was used as PCR template to generate three 145138 mutants, 145138 (Δ1-126), 145138 (Δ1-210), and 145138 (S311A), that were amplified by using mutant primers listed in Table S1, and PCR product was self-joined by In-Fusion HD cloning. The linearized expression construct was prepared by PCR using plasmid as a template, then introduced into *A. limacinum* mh0186 or Δ*145138* mutant by electroporation using Gene Pulser Xcell (750 V, 25 μF, 200 Ω). The transformants that grew on PDA-agar plate containing 0.5 mg/ml G418 were subjected to PCR screening to examine the *145138*OE line using the primer set UbiP-S and UbiT-A (Table S1).

*Purification of recombinant 145138 –* The 6×His-tagged 145138 expressing *A. limacinum* mh0186 was cultured in a 50 ml GY medium in a 200 ml Erlenmeyer flask for 4 days at 25°C with shaking at 150 rpm. Cells were harvested by centrifugation at 5000 × g for 5 min, and the supernatant was collected by centrifugation. After filtration using a 0.45 μm filter, the supernatant was applied to a Ni Sepharose 6 Fast Flow resin (GE Healthcare) equilibrated with a 50 mM Tris-HCl buffer, pH 8, containing 500 mM NaCl, and the column was washed with a 50 mM Tris-HCl buffer, pH 8, containing 500 mM NaCl and 20 mM imidazole. Recombinant 145138 was eluted from the column with 50 mM Tris-HCl buffer, pH 8, containing 500 mM NaCl and 200 mM imidazole. The lipase activity of each fraction was measured using 4MU-palmitate. The fractions showing high lipase activity were pooled and then loaded onto a HiTrap desalting column (GE Healthcare) equilibrated with a 50 mM Tris HCl buffer, pH 8, containing 150 mM NaCl for buffer exchange. Protein content was determined using the bicinchoninic acid protein assay (Nacalai Tesque Inc.) with bovine serum albumin as a standard. Similar procedure was used to prepare the recombinant 145138 (S311A) of which serine 311 was replaced with alanine. The purified recombinant 145138 (approximately 60 nmol) was applied to the protein sequence to determine the five amino acid residues at the N-terminal of the secretory form of 145138 (Nippi Inc.).

*Characterization of recombinant 145138* – The pH dependency was determined in a pH range of 4–9 using the following buffers at a final concentration of 50 mM: sodium acetate buffer (pH 4–5.5), MES (pH 5.5–7), and Tris-HCl buffer (pH 7–9). The optimal temperature was determined in the range from 15°C to 50°C. The effects of organic solvent were determined using methanol, ethanol, 2-propanol, acetonitrile, glycerol, DMSO, hexane, heptane, and acetone. The effects of metal ions were examined by adding Ca^2+^, Co^2+^, Cu^2+^, Fe^3+^, Hg^2+^, Mg^2+^, Mn^2+^, Na^+^, Ni^2+^, Li^+^, K^+^, Zn^2+^, and EDTA to the reaction mixture at 1 or 5 mM. Salt tolerance was examined using NaCl at a concentration of 0.156–2.5 M. Lipase activities were measured by using 4MU-palmitate. Phosphatase activity was measured by using *p*NP-phosphate. Calf intestine alkaline phosphatase (CIAP) was used as control for phosphatase assay. The reaction mixture was kept at 37°C for 30 min, and released *p*NP was measured by absorbance at 405 nm using a plate reader Multiskan FC (Thermo Fisher Scientific).

*Identification of ethyl ester of TF-fatty acid (TF-EE) using LC-ESI MS/MS* – One hundred pmol of TF-TG was incubated at 37°C for 30 min with rotation at 2000 rpm with 0.6 ng of 145138 in 100 μl of 50 mM Tris-HCl buffer, pH 8, containing 20 μmol of CaCl_2_ and 25% ethanol, then 400 μl of chloroform/methanol (2/1, v/v) was added in order to terminate the reaction. After centrifugation at 11,000 × g for 3 min, 100 μl of the organic phase was transferred to autoinjector vials. TF-EE was separated by liquid chromatography in the same conditions for the measurement of PC and TG, and positive Q1 scan mode (*m/z* 400 – 1100) was used for MS of TF-EE.

*Measurement of 1-acyl and 2-acyl LPC in the LB medium* – Eight nmol of 1-acyl or 2-acyl LPC was added into the 200 μl of LB medium, and was incubated at 37^o^C for times indicated in Figure S9. Twenty μl of sample was mixed with 180 μl of 95% acetonitrile with 5 mM ammonium formate, pH4.0. After centrifugation at 16,000 x g for 3 min, 150 μl of the supernatant was transferred to autoinjector vials, then 5 μl of sample was applied to LC ESI-MS/MS analysis. One-acyl and 2-acyl LPC were separated by reverse-phase chromatography using as described in ([5](#_ENREF_5)) with some modification. Briefly, InertSustain C18 was equilibrated by 55% solvent B3 (95% acetonitrile with 5 mM ammonium formate, pH4.0) in solvent A3 (distilled water with 5 mM ammonium formate, pH4.0) and was maintained for 10 min. The gradient reached 85% B for 20 min and maintained for 7 min. The gradient was returned to the starting conditions, and the column was equilibrated for 5 min before the next run.

**Supplemental References**

1. Sakaguchi, K., Matsuda, T., Kobayashi, T., Ohara, J., Hamaguchi, R., Abe, E., Nagano, N., Hayashi, M., Ueda, M., Honda, D., Okita, Y., Taoka, Y., Sugimoto, S., Okino, N., and Ito, M. (2012) Versatile transformation system that is applicable to both multiple transgene expression and gene targeting for Thraustochytrids. *Appl. Environ. Microbiol.* **78**, 3193-3202

2. Matsuda, T., Sakaguchi, K., Hamaguchi, R., Kobayashi, T., Abe, E., Hama, Y., Hayashi, M., Honda, D., Okita, Y., Sugimoto, S., Okino, N., and Ito, M. (2012) Analysis of Delta12-fatty acid desaturase function revealed that two distinct pathways are active for the synthesis of PUFAs in T. aureum ATCC 34304. *J. Lipid Res.* **53**, 1210-1222

3. Watanabe, T., Sakiyama, R., Iimi, Y., Sekine, S., Abe, E., Nomura, K. H., Nomura, K., Ishibashi, Y., Okino, N., Hayashi, M., and Ito, M. (2017) Regulation of TG accumulation and lipid droplet morphology by the novel TLDP1 in Aurantiochytrium limacinum F26-b. *J. Lipid Res.* **58**, 2334-2347

4. Okino, N., Wakisaka, H., Ishibashi, Y., and Ito, M. (2018) Visualization of Endoplasmic Reticulum and Mitochondria in Aurantiochytrium limacinum by the Expression of EGFP with Cell Organelle-Specific Targeting/Retaining Signals. *Mar. Biotechnol.* **20**, 182-192

5. Okudaira, M., Inoue, A., Shuto, A., Nakanaga, K., Kano, K., Makide, K., Saigusa, D., Tomioka, Y., and Aoki, J. (2014) Separation and quantification of 2-acyl-1-lysophospholipids and 1-acyl-2-lysophospholipids in biological samples by LC-MS/MS. *J. Lipid Res.* **55**, 2178-2192

6. Krogh, A., Larsson, B., von Heijne, G., and Sonnhammer, E. L. (2001) Predicting transmembrane protein topology with a hidden Markov model: application to complete genomes. *J. Mol. Biol.* **305**, 567-580

**Supplemental Table**

Table S1. Oligonucleotide primers used in this study.

**Supplemental Figure legends**

**Figure S1. Disruption of *145138* by homologous recombination in *A. limacinum***

(A) Diagram of the DNA construct for the disruption of *145138* by homologous recombination. Primers used and the PCR product size are indicated. (B) PCR for selection of *145138* deletion (Δ*145138*) mutants. In PCR1, the PCR products in lanes 4, 13, and 15 include 3580 bp amplicons that correspond to Δ*145138*. Δ*145138* lines selected from PCR1 were further applied to PCR2, in which a 5083-bp amplicon was generated for Δ*145138*. (C) Growth curves for WT and Δ*145138*. (D) Glucose consumption by WT and Δ*145138*. Error bars represent means ± S.D. of four separate experiments. (E) Amounts of cellular TGs. Cells of WT and Δ*145138* were harvested from 1-, 3-, 5-, and 7-day cultures. Major molecular species, TG48:0, TG54:6, TG60:12, and TG66:18, in WT (white bars) and Δ*145138* (black bars) were measured by MRM analysis using LC-ESI MS/MS. Error bars represent means ± S.D. of four separate experiments.

**Figure S2. Expression of 145138-FLAG in *A. limacinum* mh0186*.***

145138-FLAG expression construct. Thraustochytrid-derived promoters and terminators were prepared as described in ([1](#_ENREF_1)). (B) Growth curves of WT and 145138-FLAG-OE (left panel), and glucose consumption of WT and 145138-FLAG-OE (right panel). (C) Primary structure of 145138-FLAG. Alternative initiating methionine (red) was predicted by the molecular weight of 145138-expressed in *A. limacinum*. N-terminal amino acid residues of secreted 145138, as identified by the protein sequencer, are shown as white letters on a black background (D) Intracellular expression of 145138 (Full), 145138 (Δ1-126), and 145138 (Δ1-210). Cell lysates were subjected to western blotting using an antibody against DYKDDDDK. (E) Prediction of transmembrane helices of 145138 by TMHMM ([6](#_ENREF_6)).

**Figure S3. Purification and characterization of 6×His-tagged 145138**

(A) Purification of the recombinant 145138 secreted in the culture medium of *A. limacinum* mh0186. C-terminal 6×His tagged 145138 was purified using Ni-Sepharose 6 Fast Flow and HiTrap Desalting columns. Each fraction was subjected to SDS-PAGE (left panel) and western blotting analysis with an antibody against the 6×His tag (right panel). (B) Optimum pH of the recombinant 145138. Sodium acetate buffer (pH 4–5.5), MES (pH 5.5–7), and Tris-HCl buffer (pH 7–9) were used. (C) Effect of temperature on the activity of the recombinant 145138. (D) Effects of organic solvents on the activity of the recombinant 145138, with the addition of 25% organic solvents to the reaction mixtures. (E) Effects of metal ions on the activity of 145138. Several metal ions at 1 mM or 5 mM were incubated with the recombinant 145138. (F) Effects of several concentrations of Ca^2+^ and Mg^2+^ on the activity of 145138. (G) Salt tolerance of 145138. Several concentrations of NaCl were added to the reaction mixture. Lipase activities were determined using 4MU-palmitic acid as a substrate. Error bars represent means ± S.D. of three separate experiments.

**Figure S4. The structures of substrates used in this study.**

2,3-TF-TG was generated from 1,3-TF-TG by acyl migration.

**Figure S5. Transesterification reaction of 145138.**

(A) Scheme showing the hydrolysis and transesterification reactions catalyzed by 145138. Mass spectrums of TF-TG (B) and TF-EE (C) were measured using LC-ESI MS.

**Figure S6. Phospholipase A_1_ activity of 145138**

(A) Scheme for the action of 145138 on 1-oleoyl-2-[12-[(7-nitro-2-1,3-benzoxadiazol-4-yl)amino]dodecanoyl]-sn-glycero-3-PC (NBD-PC). (B) Scheme for the action of 145138 on 1-palmitoyl-2-docosahexaenoyl-sn-glycero-3-phosphocholine (PC38:6). After incubation without (C) or with (D) 145138, the mass spectra of LPC generated from PC38:6 were measured using LC-ESI MS. (E) TLC showing the hydrolysis of NBD-PA and NBD-PC. (F) The ratio of NBD-FFA and NBD-lysophospholipid (LPL) generated from NBD-PA or NBD-PC by 145138. (G) Quantification of hydrolysis extents of fluorescent lipid substrates using recombinant 145138. Fluorescence intensities of each substrate and product were quantified using a fluorescence chromatoscanner. The extent of hydrolysis of fluorescent lipids was calculated as follows: hydrolysis (%) = (peak area for NBD-LPL + peak area for NBD-FFA) × 100/(peak area for NBD-LPL + peak area for NBD-FFA + peak area for remaining substrate). (H) Phosphatase (phospholipase C) activity of 145138 examined by *p*NP-phosphate. Calf intestine alkaline phosphatase (CIAP) was used as control for phosphatase assay. Error bars represent means ± S.D. of three separate experiments.

**Figure S7. Effect of depletion/overexpression of 145138 on LDs cultured in the GY medium**

(A) LDs of the WT, Δ*145138* and *145138*OE cultured in the GY medium of which carbon source is glucose. LDs were stained by HCS LipidTOX Red neutral lipid stain. Scale bar represent 7.5 μm. (B) Diameter of LDs in WT, Δ*145138*, and *145138*OE. Diameter was measured by LAS X software. (C) Growth curves for WT, Δ*145138*, and *145138*OE in the medium containing free 18:1 as the sole carbon source.

**Figure S8. Antimicrobial activity of LPC generated by 145138**

(A) Dose dependency of the antimicrobial effect of 1-stearoyl-2-hydroxy-sn-glycero-3-phosphocholine (1-acyl LPC) and 2-stearoyl-sn-glycero-3-phosphocholine (2-acyl LPC) on gram-positive bacteria (*S. epidermidis*). (B) Antimicrobial activity of 1-acyl LPC and 2-acyl LPC on gram-negative bacteria (*P. aeruginosa*). Six nmol of 1-acyl LPC or 2-acyl LPC dissolved in ethanol was added to 150 μl of culture medium of *P. aeruginosa*. (C) Quantification of PC38:6 (substrate) and LPC22:6 (product) with or without 145138. PC38:6, LPC16:0, and LPC22:6 were quantified using LC-ESI MS/MS after incubation without (-) or with (+) 145138. (D) Direct effect of 145138 on the growth of gram-positive bacteria. *S. epidermidis* was cultured in the LB medium containing 50 mM Tris-HCl (pH 8), 20 mM CaCl_2_, and 100 ng/ml of purified 145138.

**Figure S9. Stabilities of 1-acyl and 2-acyl LPC in the medium for bacterial growth.**

Mass chromatogram of 1-acyl LPC (left) and 2-acyl LPC (right) added into the LB medium. In this condition, 2-acyl LPC is eluted from reverse-phase column before 1-acyl LPC. Acyl-migration that transfers fatty acyl chain at *sn*-2 of 2-acyl LPC to *sn*-1 generating 1-acyl LPC barely occurred in the LB medium even after 8 hour incubation.

**Supplemental Figures**

**
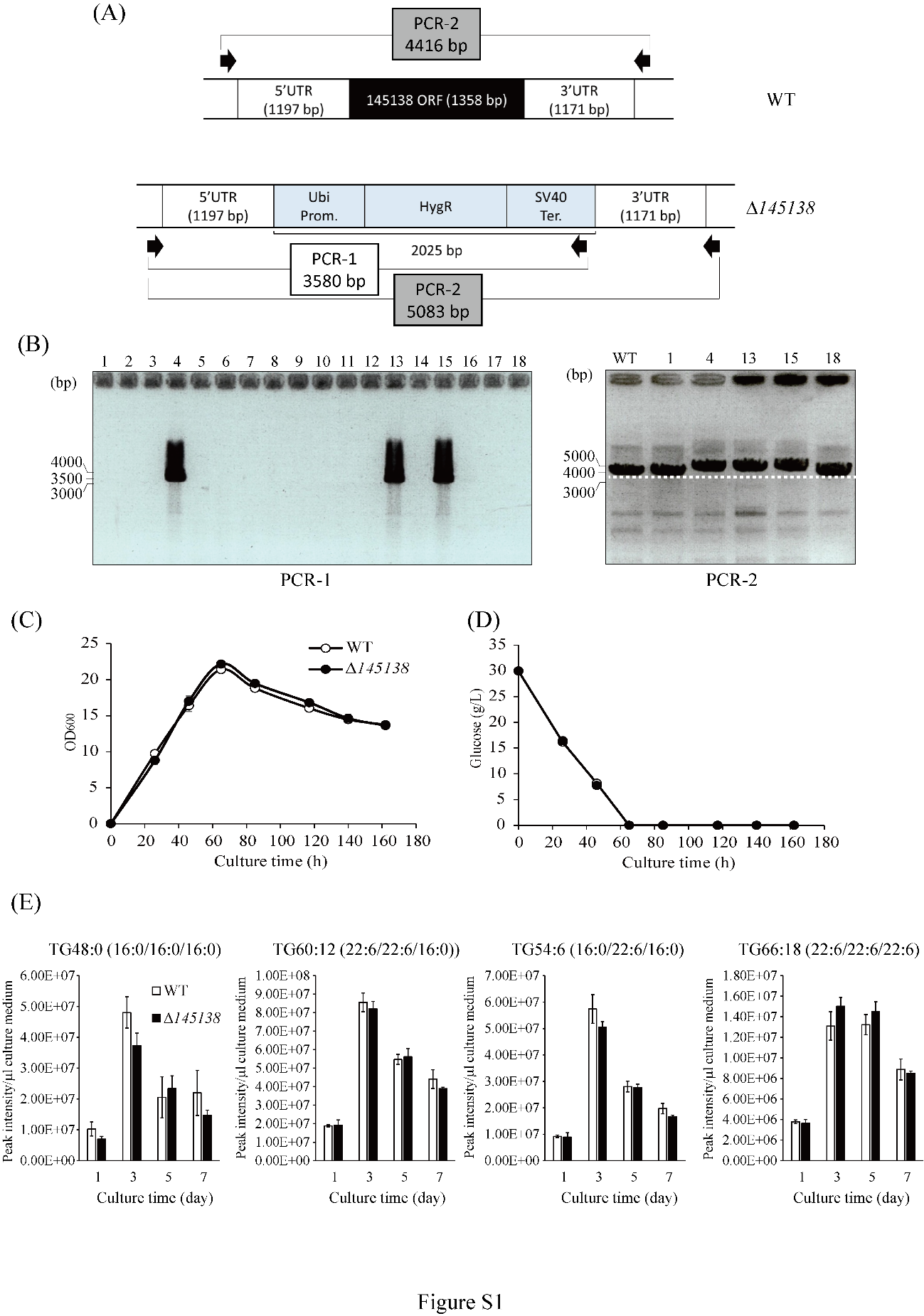
**

**
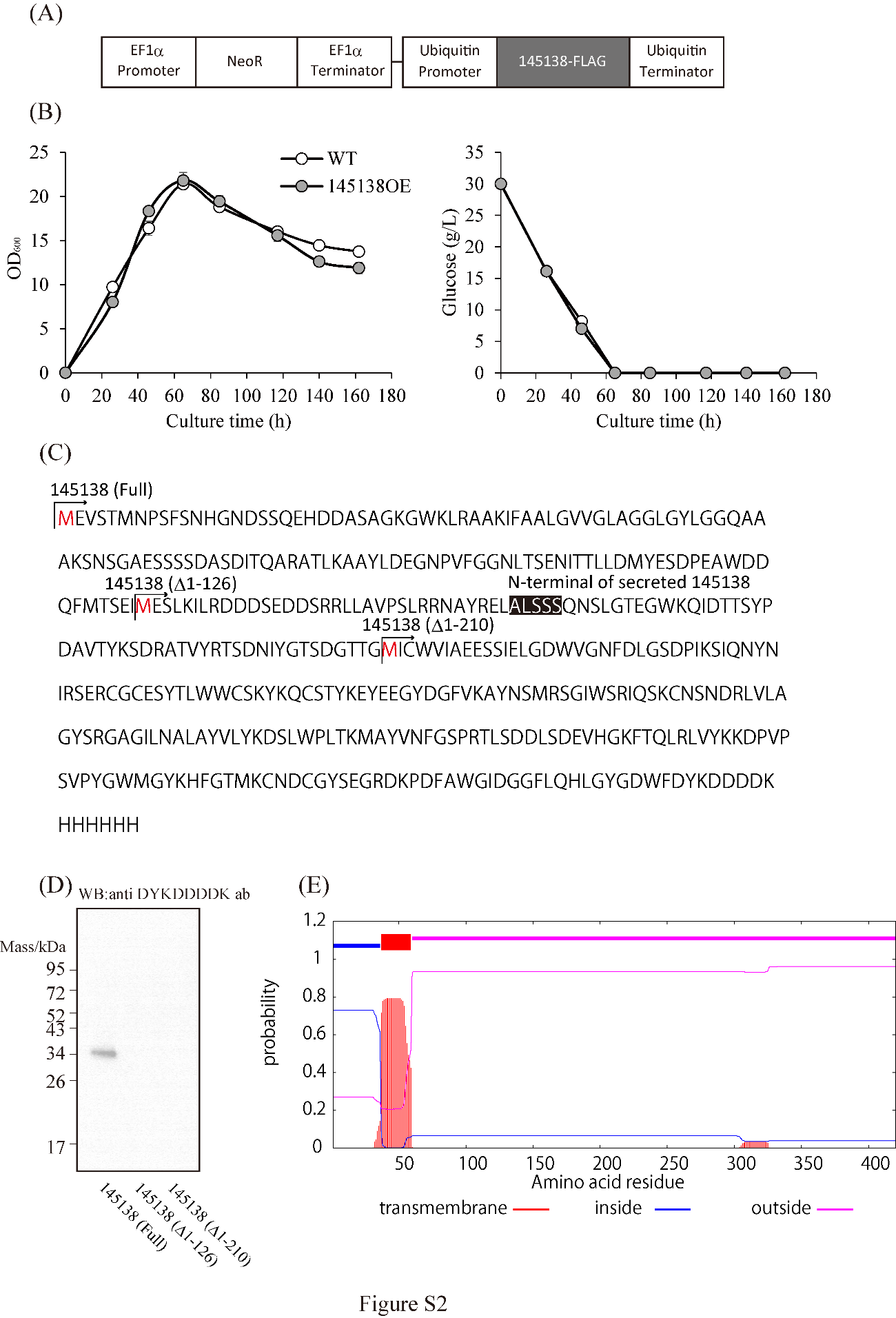
**

**

**

**

**

**
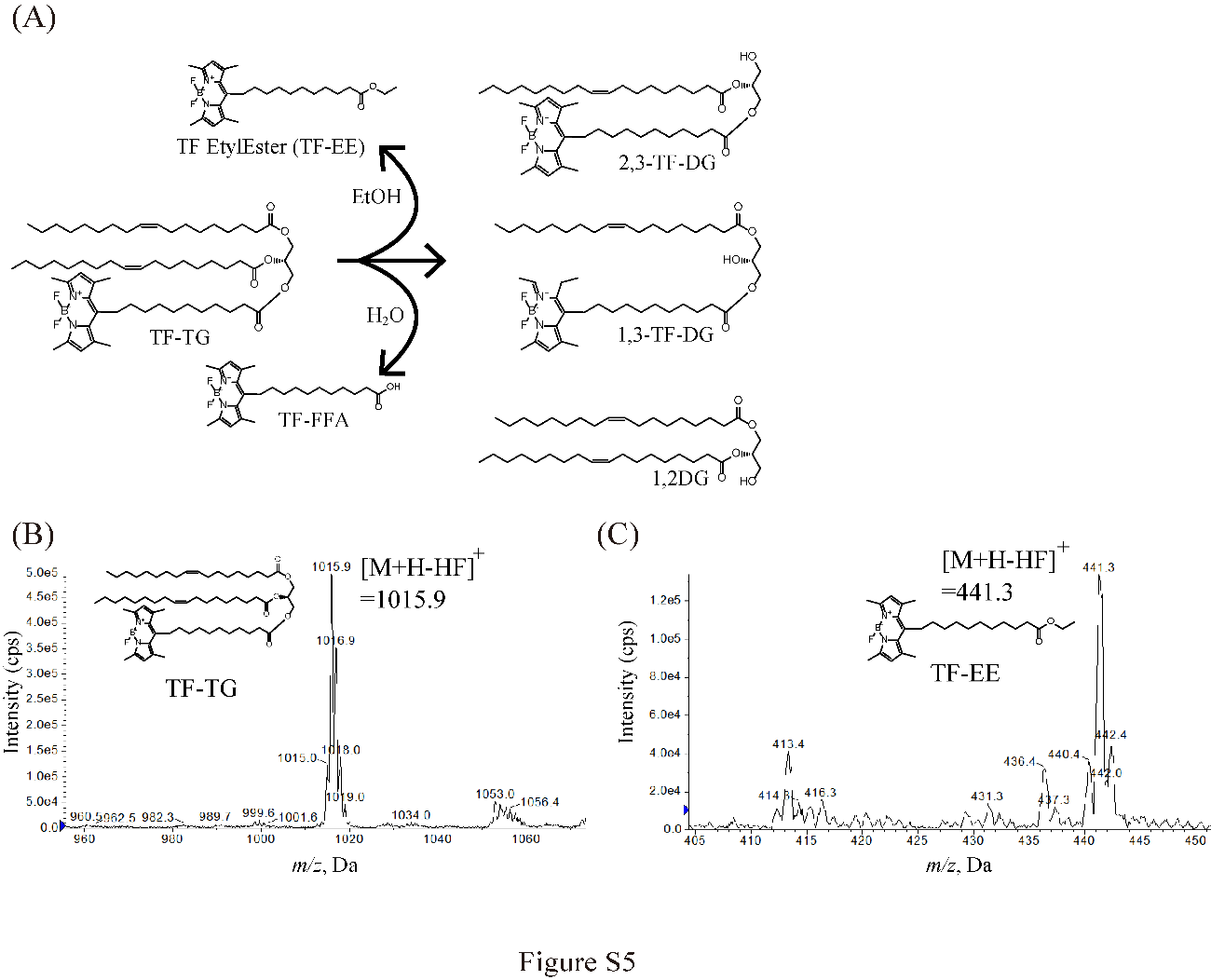
**

**

**

**
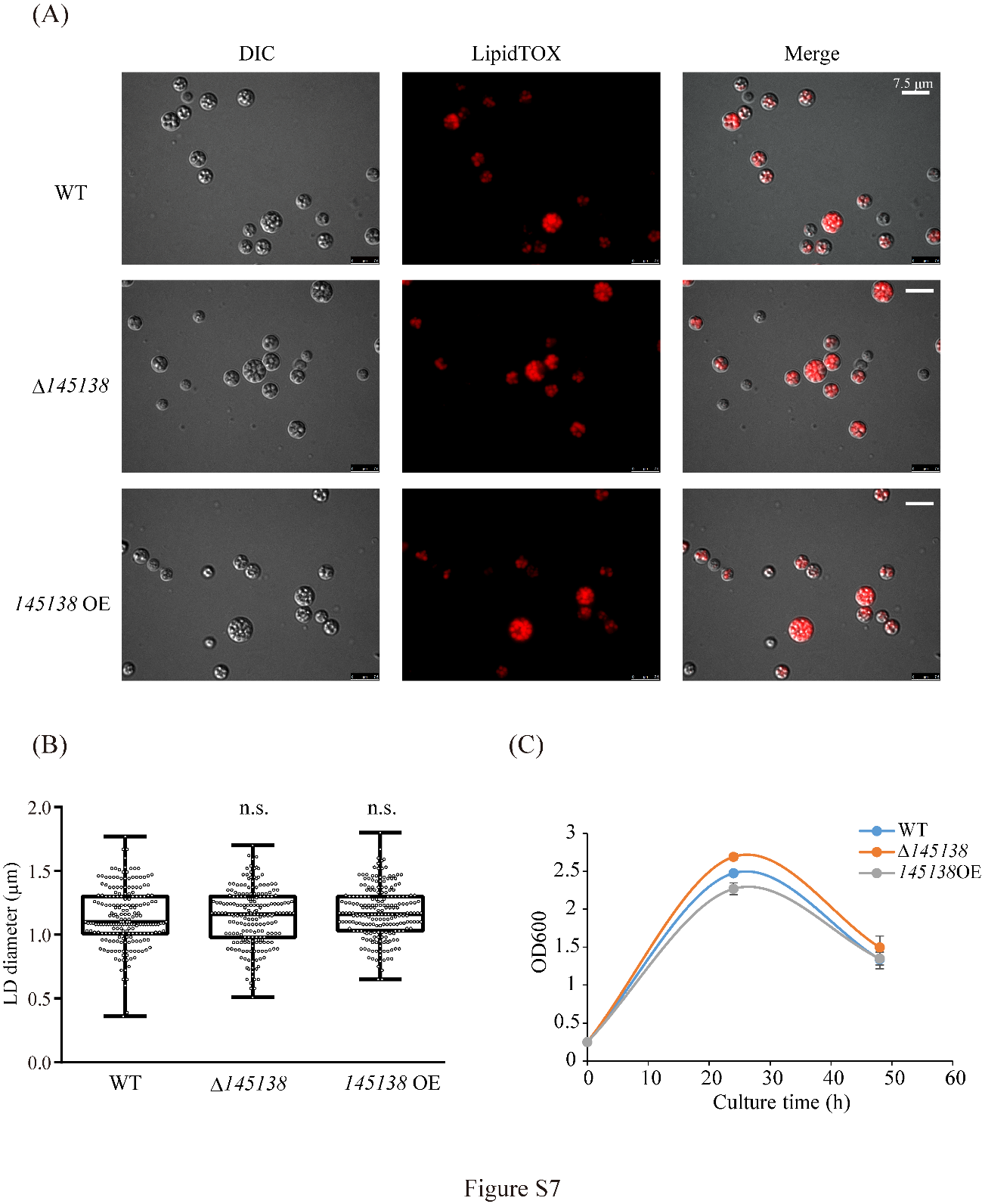
**

**
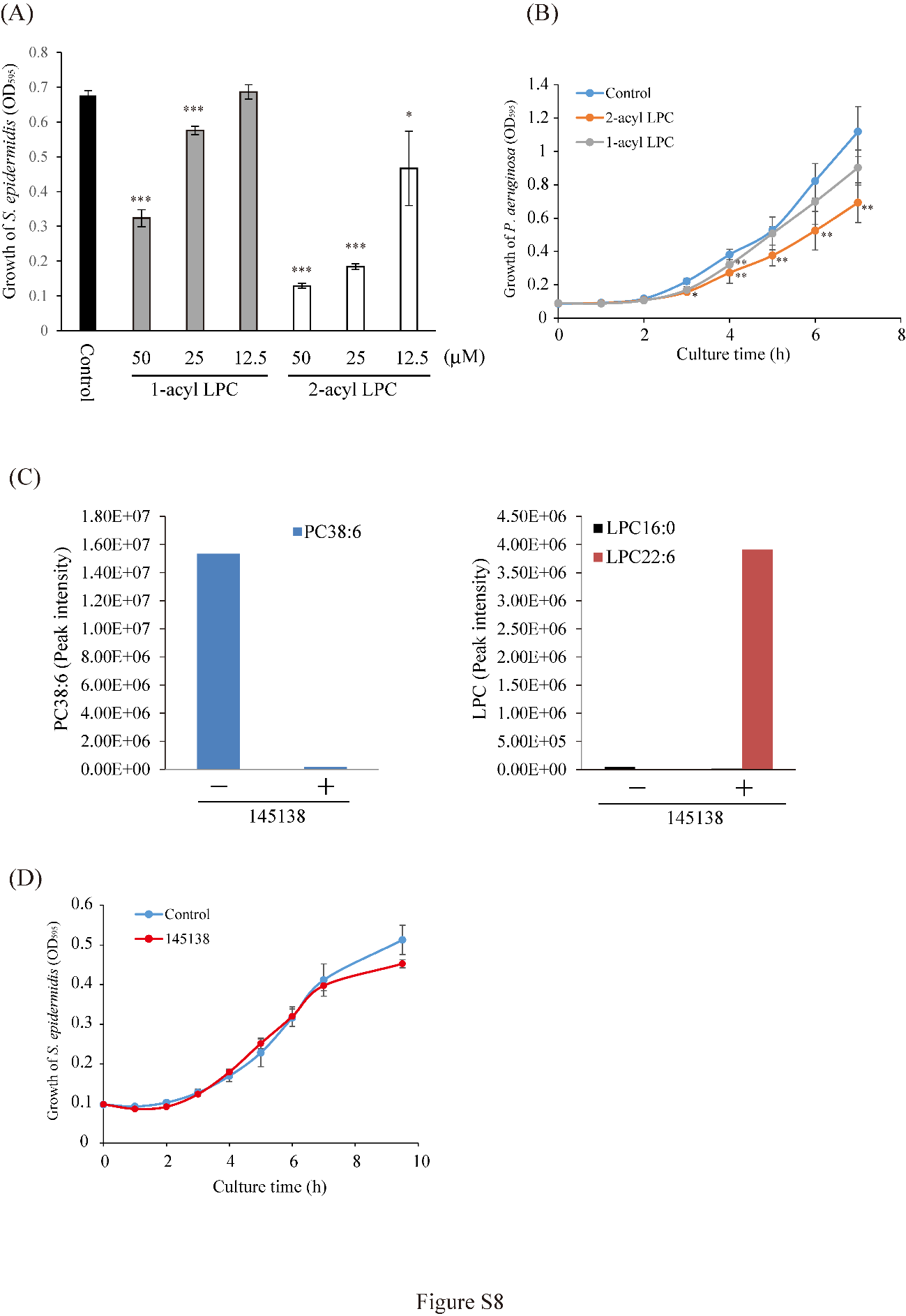
**
